# Cochlear Implant Referrals in Adults With Severe-to-Profound Hearing Loss in the United Kingdom (CIRCA-2): Protocol for a Repeat National Multicenter Retrospective Observational Study

**DOI:** 10.2196/94888

**Published:** 2026-10-13

**Authors:** Alexander Scott North, Chloe Swords, Petrina Checkland, Jessica Daniels, Oliver George Denton, Amir Habeeb, Kim Hui Lim, Isaac Jarratt-Barnham, Amar Rajgor, Bhavesh Vijay Tailor, Jeremy Wong, Jameel Muzaffar

**Affiliations:** 1 Guy's and St Thomas' NHS Foundation Trust, London, England, United Kingdom; 2 Cambridge University Hospitals NHS Foundation Trust, Cambridge, England, United Kingdom; 3 University Hospitals Birmingham NHS Foundation Trust, Birmingham, England, United Kingdom; 4 Manchester Royal Infirmary, Manchester, England, United Kingdom; 5 Queen Elizabeth Hospital, Glasgow, Scotland, United Kingdom; 6 Barking, Havering And Redbridge University Hospitals NHS Trust, London, England, United Kingdom; 7 Royal Hallamshire Hospital, Sheffield, England, United Kingdom; 8 St George's Hospital, London, England, United Kingdom; 9 Population Health Sciences Institute, Newcastle University, Newvastle, England, United Kingdom; 10 John Radcliffe Hospital, Oxford, England, United Kingdom; 11 Department of Applied Health Sciences, University of Birmingham, Edgbaston, Birmingham, England, B15 2TT, United Kingdom, 44 7447065968

**Keywords:** cochlear implants, referral pathways, health inequalities, national audit, observational study, United Kingdom

## Abstract

**Background:**

Cochlear implantation (CI) is a highly effective intervention for adults with severe-to-profound hearing loss, yet uptake across the United Kingdom remains low despite clear National Institute for Health and Care Excellence (NICE 2019) guidance and well-established clinical benefits. The original Cochlear Implant Referral Criteria Audit (CIRCA-1) identified marked inequities in access to CIs in 2022, with lower referral rates among older adults, males, and individuals from socioeconomically deprived areas, alongside individuals from minority ethnic backgrounds being less likely to be informed of their eligibility for referral. Since then, national awareness initiatives and the expansion of the existing local “CI Champions” scheme have aimed to address these inequities, improve awareness of CI candidacy, and improve the quality of discussions with patients about options for CI referral. This repeat assessment aims to assess the success of these interventions to identify improvements in access to CIs as well as persisting barriers.

**Objective:**

CIRCA-2 is a national, multicenter, retrospective observational audit designed to re-evaluate adult CI referral pathways and measure progress since the first audit cycle.

**Methods:**

The study will include adults assessed in secondary-care ear, nose, throat, and audiology departments between July and December 2025 whose audiometric thresholds meet NICE TA566 criteria. Participating centers will identify eligible patients using the British Cochlear Implant Group Crystal Report within Auditbase, and anonymized demographic, audiometric, and service-level data will be extracted. The primary outcomes are the proportions of patients meeting NICE TA566 criteria who were informed of their eligibility and referred for CI assessment, with comparisons made to CIRCA-1 data. Secondary analyses will examine adherence to national standards and predictors of discussion and referral using multivariable modeling. In addition, the study will assess the number of patients within criteria for the Comparing Cochlear Implants With Hearing Aids in Adults (COACH) trial and those meeting CI criteria in one ear only.

**Results:**

Retrospective data for the period July 1 to December 31, 2025, were collected between January and March 2026. Eighty-three sites submitted data relating to 15,060 patients. Data analysis is projected to be complete by October 2026, with publication of final results expected in early 2027.

**Conclusions:**

By quantifying change over time, CIRCA-2 will provide an updated national picture of CI referral practice, highlight any persisting barriers, and inform future strategies to promote equitable access to CIs.

## Introduction

### Background

Hearing loss is one of the leading causes of disability worldwide, affecting more than 430 million people, a figure projected to exceed 700 million by 2050 with population aging [1]. Severe-to-profound hearing loss is associated with substantial reductions in communication, educational and employment opportunities, social participation, and quality of life, while also increasing the risks of depression, social isolation, and cognitive decline [1,2]. In the United Kingdom, approximately 1.2 million individuals are affected by severe-to-profound hearing loss, a condition projected to become a top 10 health burden by 2030, with an estimated annual cost of £38.6 billion (US $51.1 billion [£1=US $1.32] as of October 6, 2026) to the UK economy [3,4].

Cochlear implants (CIs) are transformative medical devices that provide electronic hearing to individuals with severe-to-profound hearing loss and have been shown to significantly improve quality of life [5]. They have been demonstrated to be a cost-effective intervention across multiple different health care systems [6,7]. According to recent estimates, around 25,000 children and adults in the United Kingdom are CI users [8]. Despite the well-documented benefits of CI, it is estimated that only 5% to 10% of potentially eligible patients currently receive a CI [9,10]. Numerous studies have demonstrated that only a minority of adults who fulfill referral or candidacy criteria are ultimately assessed for implantation internationally [9-12]. Barriers exist at multiple levels of the health care pathway, including limited awareness among health care professionals and patients, variation in referral practices, geographical inequity, socioeconomic disadvantage, cultural perceptions of hearing loss, and inconsistent access to specialist implant centers [12]. Consequently, improving equitable access to CI has become an important international priority in hearing health care.

A comprehensive national assessment of referral patterns was undertaken for the first time in 2022: the Cochlear Implant Referral Criteria Audit (CIRCA-1) study, which included 36 UK secondary care centers. In this study, 6171 adults who met audiometric criteria were identified, with only 35.7% being informed of their eligibility and just 9.7% being referred for CI assessment [13]. The CIRCA study also highlighted inequities in access, with lower referral rates among individuals from socioeconomically deprived areas, ethnic minority groups—particularly Asian and Black individuals, older adults, males, and those residing in London and the North [13]. Since its publication, the results of CIRCA-1 have been disseminated through national bodies, including the British Cochlear Implant Group (BCIG) and the British Association of Audiology (BAA). Nationwide awareness initiatives and educational content alongside expansion of the BAA “CI Champion” scheme, a job role where an audiologist has dedicated time to audit local referrals and work to improve referral pathways with a “mentor” at an implant center. However, there has not been an interval assessment on whether these interventions have influenced referral patterns or increased awareness of the National Institute for Health and Care Excellence (NICE) 2019 guideline changes [14]. Although national adult CI activity has begun to recover after the COVID-19–related dip in 2020 to 2021, recent figures (2024‐2025) show only a modest increase of around 6% compared with pre-TA566 levels, which is substantially lower than the 70% rise predicted by NICE when moving from TA166 to TA566. This discrepancy further highlights the need for a repeat national audit to evaluate progress, measure adherence to current standards, and identify persistent barriers.

In addition to individuals who meet current NICE criteria [14], there remains interest in the role of CI for those who fall outside current thresholds. The ongoing COACH (Comparing Cochlear Implants With Hearing Aids in Adults) trial is an ongoing randomized trial comparing hearing aids to CI for patients with hearing at 70 decibels or worse in 2 frequencies between 500 and 4000 Hz in both ears alongside speech perception <60% on Arthur Boothroyd Word Test [15]. The COACH trial criteria are more relaxed than current NICE guidelines, and adoption of these would make more patients eligible for CI and have corresponding implications on workforce planning and service design. The extent of this effect is uncertain, and a report on the proportions of additional patients meeting COACH entry criteria would be informative and will be performed alongside the primary reanalysis using existing NICE guidance. This will provide real-world context on the potential increased demand and capacity implications of potentially lowering the threshold of audiometric criteria for CI candidacy. As such, this study will help re-explore existing barriers that patients face accessing CIs and assess whether current interventions have been impactful. This will help direct future improvements to enable equitable access to CIs and the benefits they provide.

Understanding where potentially eligible patients are lost within referral pathways is fundamental to improving access to CI. National clinical audits provide an opportunity to benchmark practice against established standards, identify unwarranted variation between health care providers, evaluate the impact of quality improvement initiatives, and support evidence-informed service planning. Although referral criteria differ between health care systems, the underlying challenges of delayed identification and inconsistent referral are common internationally, making reproducible audit methodologies valuable beyond individual countries. This protocol provides a framework for such an assessment that can be implemented in other health care systems beyond the United Kingdom.

### Aims and Objectives

#### Aim

The aim of this study is to reassess referral patterns for patients suitable for CI in the United Kingdom, building on findings from the original CIRCA study.

#### Objectives

The objectives of this study are to:

1. Measure the proportion of adults meeting current NICE TA566 criteria [14] for CI assessment on audiometric testing who are referred for assessment, and compare these proportions with those from the CIRCA-1 study.
2. Measure the proportion of adults meeting current NICE TA566 criteria [14] for CI assessment on audiometric testing who are told that they are eligible for assessment, and compare these proportions with those from the CIRCA-1 study.
3. Identify predictors of discussion of implantation and referral for assessment, and assess whether the strength or pattern of these predictors differs from those identified in the CIRCA-1 study [13].
4. Report adherence to BCIG and BAA recommendations that each site should have a CI champion, and evaluate whether uptake has improved compared with the original study.
5. Measure the proportion of adults who meet the pure-tone audiometric thresholds used in the COACH trial to estimate the service implications of a potential future relaxation of CI eligibility criteria [15].

## Methods

### Study Design

This study is a national, multicenter, retrospective observational audit of practice. The overall study workflow is demonstrated in Figure 1. This study will be reported in accordance with the STROBE (Strengthening the Reporting of Observational Studies in Epidemiology) guidelines.

**Figure 1.**
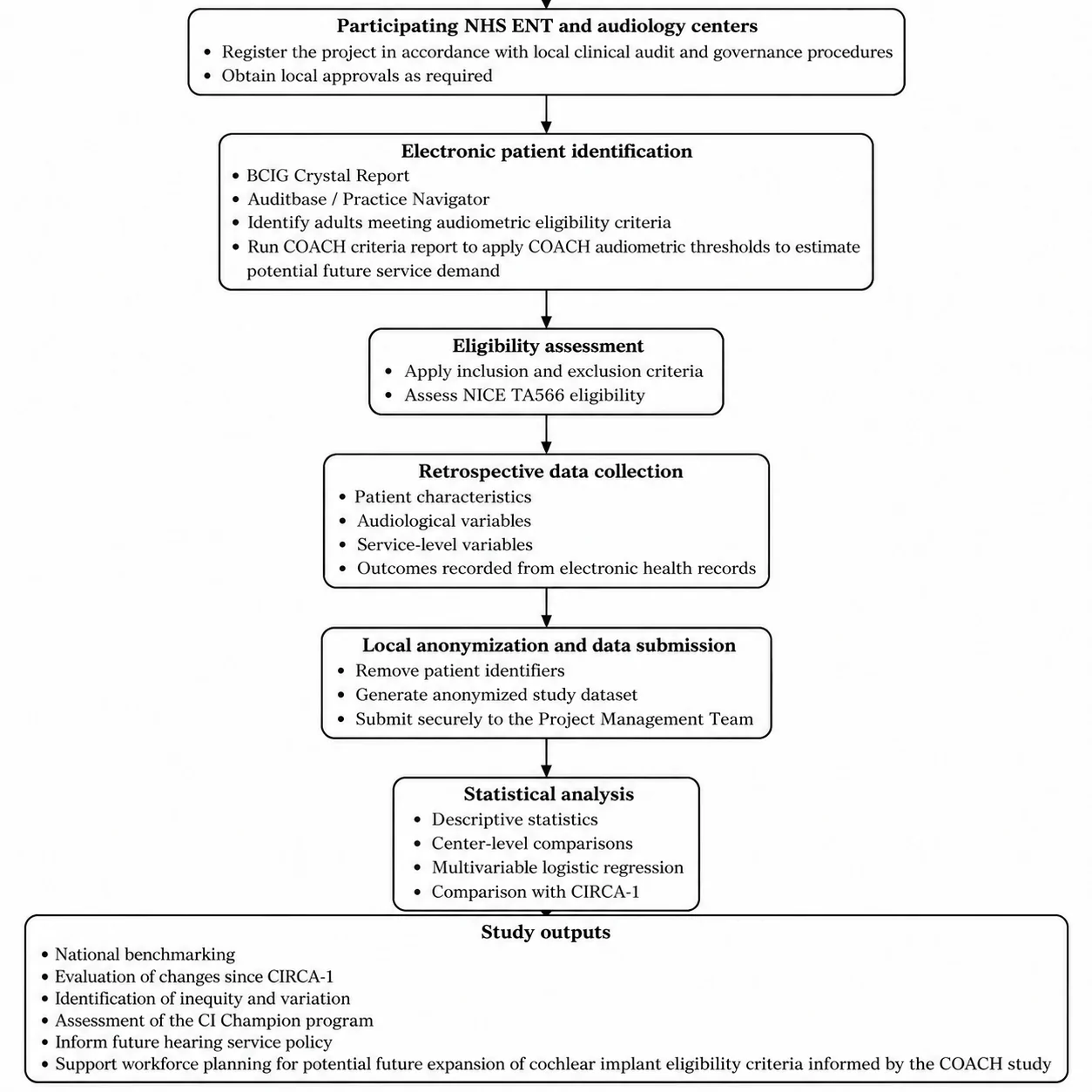
Overview of the CIRCA-2 study workflow. Participating NHS ENT and audiology centers register the project according to local governance procedures before identifying potentially eligible adults using the BCIG crystal report within auditbase/Practice Navigator. Following application of the eligibility criteria, retrospective data are extracted from electronic health records, anonymized locally, and submitted securely to the Project Management Team for statistical analysis. The audit will generate national benchmarking data, evaluate changes since CIRCA-1, identify variation in referral practice, assess the impact of the CI Champion program, inform hearing service policy, and support workforce planning for potential future expansion of CI eligibility criteria informed by the COACH study. BCIG: British Cochlear Implant Group; CI: cochlear implant; CIRCA: Cochlear Implant Referral Criteria Audit; COACH: Comparing Cochlear Implants With Hearing Aids in Adults; ENT: ear, nose, and throat; NHS: National Health Service; NICE: National Institute for Health and Care Excellence.

### Setting

A multicenter study will be conducted in units providing ear, nose, and throat (ENT) and audiology services within the NHS across the United Kingdom. Any unit with ENT and audiology services will be eligible to recruit patients if they use Auditbase or Practice Navigator software. Auditbase is the most common database in UK audiology departments. The second most common, Practice Navigator, has been progressively phased out since 2022 and will be used in fewer sites than when the original study was conducted. The increased use of Auditbase by sites across the United Kingdom will facilitate engagement from an increased number of sites compared with CIRCA-1 and will likely facilitate a larger sample size and more representative results. Since the original audit, there is now a method to perform searches in Practice Navigator, enabling inclusion of centers that use the 2 largest audiology databases in the United Kingdom.

### Collaboration and Delivery

The study will be delivered by INTEGRATE, the UK ENT Trainee Research Collaboration [16], supported by INDICIA and the CI Champion Scheme. This collaborative structure consists of a central Project Management Team (PMT), with invitations to all eligible sites for audiologists, ENT clinicians, or audiovestibular medicine physicians to join as collaborators on this project. This collaborative framework mirrors the CIRCA-1 audit, which is outlined in detail below.

### Study Setup

INTEGRATE will identify regional leads for each UK region who will, in turn, help recruit local leads for each hospital. Each hospital will have a local audiology and/or ENT resident doctor lead and/or audiovestibular medicine clinician lead. The local lead will collaborate with the local audiology team and then register the study with the audit department locally before data collection begins. For units with more than 100 eligible patients, the local lead may choose another collaborator to divide the data capture work with, with a further collaborator granted for each additional 100 patients. An effort will be made to rerecruit all original CIRCA sites.

### Participant Characteristics

#### Sample Size

The final sample size will depend on the number of participating centers. Based on the CIRCA-1 experience and the average case volumes (mean ~182 eligible patients per site per 6 mo), we anticipate data from more than 50 centers, yielding approximately 9000 patients nationally. Increased recruitment allows more robust and detailed analysis compared with the first cycle and can help validate the results from 2021.

#### Patient Identification

At each participating unit, the local ENT or audiology leads will run a search in Auditbase or Practice Navigator to identify a list of patients for inclusion in the study. The search will highlight eligible adults with an audiogram meeting current NICE criteria for CI [9]. A minority of patients who were unable to complete pure-tone audiometry will not be captured by this study regardless of whether they were referred for CI assessment based on their clinical assessment. The search terms have been predefined by the PMT and will be based on the BCIG Crystal Report. The Crystal Report is software that a user can use to search Auditbase to identify patients meeting defined criteria, in this instance with an audiogram meeting NICE criteria for a CI. Sites using Practice Navigator would need to adopt a more manual search technique as the Crystal Report is not compatible. The precise Auditbase and Practice Navigator search methodologies are available in Multimedia Appendix 1.

The search will be conducted to identify patients with audiograms completed between July 1, 2025, and December 31, 2025. The local lead will interrogate the ENT and audiology clinical records to confirm full eligibility against the following inclusion and exclusion criteria.

### Inclusion Criteria

Patients will be included if they meet the following criteria:

- Adult patients (18 y and older); and
- The following criteria based on audiometric testing:
  - NICE criteria TA566: unaided pure-tone audiometric threshold equal to or greater than 80 dB HL at 2 or more frequencies (500, 1000, 2000, 3000, or 4000 Hz) in both ears.
  - COACH criteria: unaided pure-tone audiometric threshold equal to or greater than 70 dB HL in both ears at 2 or more frequencies.
- Both new and follow-up patients will be included, with no limitations based on visa status.

### Exclusion Criteria

Patients will be excluded if they meet the following criterion:

- Patients with a clinical suspicion of, or further audiological testing consistent with, nonorganic hearing loss.

### Study Processes and Methodology

Project registration and governance processes will follow the same structure as the CIRCA-1 audit, which is outlined below.

#### Project Registration

Site leads will identify a senior individual at the hospital trust who will take responsibility for the project at the local hospital. This person will be known as the “lead clinician.” This is likely to be an ENT surgeon, senior audiologist, or audiovestibular medicine clinician, though if local audit registration rules allow other groups, such as clinical scientists, to be responsible clinicians for audit, then they may be the “lead clinician” at the discretion of the local audit department. Local site leads must register this project with the local Clinical Governance Department responsible for the conduct of local audit prior to data collection and submission of data to the PMT.

#### Data Anonymization Process

The PMT will not request or be provided with the names, addresses, NHS numbers, medical record numbers, or identifiable data for any patients. Patient postcodes will be used locally to generate distance to the nearest CI center and indices of multiple deprivation scores. Distance to the closest center and index of multiple deprivation rank/decile (not postcodes) will then be submitted to the PMT. Local teams will calculate patient age at the date of the first pure-tone audiogram in criteria. Age at the time of index audiological assessment (not date of birth) will be submitted to the PMT.

Reporting and analysis will not identify individual cases in any subsequent reports, presentations, or publications. Data flow will be one way, from the Data Discloser to the Data Receiver (ie, PMT). There will be no traceability from the Data Receiver database to local records. If any identifiable data are received, the files will be deleted, and the site will be informed.

### Outcome Measures

The primary outcome is to determine the factors associated with referral after completing audiometric evaluation for implant assessment. The secondary outcome is to identify the factors impacting whether health care professionals have discussed the option of referral after completing audiometric evaluation with the patient.

Based on patient hospital records, site leads will determine whether a CI referral was discussed with the patient. This will form the basis of the primary (whether a patient was referred) and secondary (whether a patient was informed that they were eligible for referral) outcomes. The different types of discussions will be coded as follows:

- CI 1=referred for CI: Patients who have already been referred to an NHS CI implant department.
- CI 2=discussed and not referred: unsuitable/outside of criteria: Patients who are not suitable for a referral at present, for example, anomaly in data, BAHA more appropriate, acute illness, or end of life.
- CI 3=discussed and not referred: discussed and further assessment needed: patients who may need further appointments in audiology/ENT before an onward referral, for example, trial of new optimized hearing aids or incomplete audiogram.
- CI 4=discussed and not referred: patient declined following documented discussion.
- CI 5=CI to be discussed: No CI discussion documented/appointment scheduled to discuss CI.
- CI 6=patient already has a CI.

Local leads will extract data from clinical and audiological records to identify potential predictors of referral for CI assessment for eligible patients and to determine which factors may be positive or negative predictive factors. These can be subdivided into patient and center predictors.

Patient predictors will include the following: age, sex, ethnicity, indices of multiple deprivation: rank and decile (measured by postcode), languages spoken (including whether English is the primary language), audiometric data (including severity of hearing loss), medical comorbidities, physical disability (defined according to *International Classification of Diseases* codes “reduced mobility,” dependence on a wheelchair, dependence on other enabling machines or devices, or paralysis, excluding those with temporary changes), and cognitive disability (record of “dementia/cognitive decline/AMTS<8/MMSE<24”).

Center predictors will include the following: colocation (or not) with a CI center; distance to the closest CI center; socioeconomic status of the center’s region; CI Champion presence (or not); CI Champion duration in role (months); CI Champion proportion of nonclinical time allocated to this role within their work schedule; in the calendar year of 2025, did the CI Champion consistently undertake the 1 hour of protected time each week? (5-point Likert scale: Never-Always); presence of speech testing availability at the local center; frequency of running of the Crystal Report or audit of local referral practice; changes in practice since CIRCA-1 if the site took part; and which version of Auditbase or Practice Navigator the site uses (this is relevant as newer versions have built-in features and prompts that could infer an increased likelihood of discussion and/or referral).

### Data Management

#### Data Pseudonymization and Prevention of Duplicate Records

NHS numbers were processed locally using a provided offline spreadsheet and an MD5 hash function to generate a consistent 20-character study linkage code. The same NHS number therefore generated the same linkage code at different participating sites, allowing potential duplicate records to be identified without transmitting the NHS number itself to the PMT. NHS numbers and other direct patient identifiers remained at the local site and were not included in the centrally submitted dataset.

#### Source Documents

The patient record (both medical and audiology notes and letters) will provide the majority of the data. The electronic medical record systems vary between trusts.

#### Data Collection Tools

Anonymized data will be locally entered into predefined data extraction forms. Data extracted will include the following: outcome of CI discussion (if any), date of audiogram, aided AB phoneme score performed (yes/no), aided AB word speech testing score range, patient’s spoken languages and main language, age, sex, ethnicity, medical comorbidities, learning disability, cognitive impairment, visual impairment, history of meningitis, physical disability, audiometric data, and details of current hearing aids (if any). The quality of the data entered will be controlled by limited data entry, drop-down options, and predefined data formats. Range checks for chosen fields will automatically appear where data points are outside of a prespecified range. Ages are automatically extracted from Auditbase. The site lead will need to calculate the index of multiple deprivation score/rank and distance between the hospital and patient postcode using a mapping site.

#### Data Handling and Record-Keeping

Anonymized data will be submitted via an NHS email account to the PMT. No patient-identifiable information will be sent or stored by the PMT.

Anonymized data may be made available to applicants who submit a project proposal to the PMT and whose proposal receives approval from the PMT, expert advisory group, and INTEGRATE main committee.

#### Archiving

Local data forms should be kept according to local data governance for 12 months following submission to allow appropriate local analysis and scrutiny from the PMT. At the end of this period, local data forms should be destroyed/deleted in line with local data governance policy.

The anonymized collated data will be held by the PMT for at least 5 years to allow for appropriate scrutiny and reanalysis.

### Statistics and Data Analysis

Descriptive statistics will be used to summarize patient, audiometric, and site characteristics. Categorical variables will be presented as frequencies, percentages, and 95% confidence intervals, with the number of patients contributing to each analysis clearly stated. Continuous variables will be presented as means with SDs or medians with IQRs, according to their distribution. The number and proportion of patients classified within each CI outcome category will be reported, together with the relevant denominator.

The prespecified primary outcomes are: (1) documented discussion of CI and (2) referral for CI assessment. These will be analyzed as binary outcomes using multilevel logistic regression with a random intercept for hospital to account for clustering of patients within sites. Results will be presented as adjusted odds ratios with 95% confidence intervals. Candidate predictors will be selected a priori on the basis of the CIRCA-1 analytical framework and clinical plausibility. When CIRCA-1 and CIRCA-2 data are analyzed together, audit cycle will be included as an indicator variable, and interaction terms will be used to examine whether selected associations differ between audit cycles.

The 6-category CI1 to CI6 classification will provide a more detailed secondary description of the referral pathway. Frequencies and percentages will be presented for each category. Where category sizes permit, associations between patient- and hospital-level characteristics and the detailed pathway outcome will be explored using multinomial logistic regression, with CI1 or another clinically appropriate category specified as the reference group. Results will be presented as relative risk ratios with 95% confidence intervals. Patients already under CI assessment and patients already implanted will be reported separately and excluded from the primary binary outcome models.

For binary regression models, model performance and adequacy will be assessed using calibration plots, the area under the receiver operating characteristic curve, residual and influence diagnostics, and likelihood-based measures of fit. For multilevel models, between-hospital variance and the intraclass correlation coefficient will also be reported. Multinomial model fit will be examined using likelihood ratio tests and information criteria. Collinearity between predictors and the functional form of continuous variables will be assessed before final model specification.

As the purpose of the analyses is explanatory and comparative rather than the development of a clinical prediction model, the principal models will be fitted using the full available dataset. Where predictive performance is reported as a secondary analysis, internal validation will use bootstrapping or cross-validation rather than a single training/test split. The contribution of individual predictors will be assessed using adjusted effect estimates, confidence intervals, and likelihood ratio tests.

### Missing Data

The PMT will run range, logic, and completeness checks on all submissions. Data queries will be sent to local leads to resolve discrepancies or complete missing critical fields (unique identifier, age, sex, audiometric thresholds used to define eligibility, and the primary outcome—outcome of CI eligibility discussion). Patients with untested frequencies on their audiograms will not be excluded, provided the index audiogram is sufficient to meet current NICE criteria. Records missing the primary outcome after query resolution will be excluded from the corresponding primary analysis but may contribute to secondary analyses where applicable.

Missingness will be described for each variable. Complete-case analysis will be used where missingness is limited; multiple imputation will be considered for predictors with more substantial missing data where its assumptions are considered reasonable. Sensitivity analyses will include exclusion of records with missing primary outcome data and exclusion of potentially duplicate records.

### Duplicate Data

Individuals treated at more than one trust will have records linked through identification of a common unique code generated from the anonymized NHS number.

### Comparison Analysis

To assess progress since the original CIRCA audit, outcomes from the current reaudit (CIRCA-2) will be directly compared with those from CIRCA-1 (2021 data collection).

Primary comparisons include the proportion of patients meeting NICE criteria who were informed of their eligibility and the proportion referred for CI assessment.

Secondary comparisons include adherence to NICE TA566 standards [14] by region and unit; presence and role of CI Champions. This is predicted to be greatly expanded compared to CIRCA-1, and this further analysis will provide insight into the impact of this scheme on discussions and referrals; proportion of patients meeting COACH pure-tone audiometric thresholds [15]; and patient or center factors that are predictors of discussion about CI eligibility and/or referral to a CI center.

Statistical testing may include chi-square or Fisher exact tests for categorical variables (eg, proportion referred vs not referred); *t* tests or Mann-Whitney *U* tests for continuous variables, depending on distribution; and/or multivariable logistic regression models including an indicator variable for audit cycle (CIRCA-1 vs CIRCA-2) to explore whether predictors of referral and implantation have changed over time.

### Subgroup Analyses

Data will be captured on the number of patients potentially within criteria (by center and region), the proportion with whom potential implantation was discussed, the proportion subsequently referred for assessment, and the proportion ultimately implanted. The proportion of potential candidates for each unit will be calculated by center and region.

As this is a reaudit, these figures will be compared directly with those from the original CIRCA audit to evaluate changes over time. Multiple regression analysis will again be used to identify predictors of discussion and referral. These predictors will include patient demographics (eg, age, gender, ethnicity, socioeconomic deprivation), geographical factors (region, presence of a colocated CI center, distance to the nearest CI center), and service factors (presence and role of a CI Champion, as recommended by BCIG and BAA). Where possible, shifts in the influence of these predictors will also be compared with those identified in the original audit.

### Ethical Considerations

#### Ethical Approval

CIRCA-2 was classified as a clinical audit rather than research using the UK Health Research Authority “Is my study research?” decision tool and the UK Policy Framework for Health and Social Care Research [17-19]. The output from this process is available in Multimedia Appendix 2. As the project is not classified as research, Health Research Authority Approval and NHS Research Ethics Committee review are not required. Each participating center is nevertheless required to register the project through its local clinical audit/clinical governance procedures and obtain all necessary local organizational approvals before data collection.

This project will report on the outcomes of investigative management that have already occurred. As there will be no deviation from the usual standard of care, formal ethical approval is not required. Data will be anonymized by the local site lead prior to submission. Due to this, no formal informed consent is required.

This audit will report on the outcomes of investigative management that have already taken place. All data will be anonymized, and no patients will be identifiable in any subsequent reports, presentations, or publications. As such, consent from individual patients will not be required.

#### Anticipated Benefits

If participants appear to meet potential eligibility criteria and CI has not previously been discussed with them, they may be contacted by their local clinical team using a standardized invitation letter. The PMT will not contact patients directly; all patient communication will be undertaken at the discretion of the local site. The PMT does not endorse direct referral for implantation based solely on audit findings. Any onward referral should follow appropriate clinical consultation and, where available, additional speech testing.

#### Potential Hazards

There are no significant potential hazards to this retrospective study. If patients have had test results that would potentially be within criteria for CI assessment, but this was not discussed, then this may cause distress, though as part of a clinical Duty of Candor, this should be discussed with them.

### Authorship Policy

#### Criteria for Inclusion as a PubMed-Citable Collaborator

Authorship will be in line with INTEGRATE policy on multicenter collaborative projects. Each center will have a named ENT and Audiology Lead. They will be eligible for collaborative coauthorship of publications that result from the project. For every additional 100 patients identified at the site, an extra ENT and Audiology collaborator can contribute.

#### Criteria for Acknowledgments

All individuals contributing to data collection will be acknowledged in any subsequent presentations and publications.

## Results

### Study Timeline

The study launched January 1, 2026, to allow the collection of retrospective data for patients tested between July 1, 2025, and December 31, 2025. Final submission of data was at 3 months from the launch date, with a deadline of March 31, 2026. This was extended to May 31 to maximize the number of submitting sites. Data analysis is projected to be complete by October 2026. The publication of final results is projected in early 2027.

### Preliminary Observations

Eighty-three sites from all 4 devolved nations of the United Kingdom have submitted data relating to 15,060 patients with an audiogram meeting NICE criteria for a CI. Planned data analysis is underway and due to be complete by October 2026.

## Discussion

CI remains a transformative intervention for adults with severe-to-profound hearing loss, yet persistent variation in referral patterns limits equitable access in the United Kingdom. The original CIRCA audit identified substantial disparities associated with age, ethnicity, and socioeconomic status, prompting national initiatives to promote awareness and establish local “CI Champions.” CIRCA-2 will re-evaluate referral practice following these efforts, providing a timely reassessment of access, awareness, and adherence to NICE guidance.

As a national multicenter retrospective observational audit, CIRCA-2 offers a pragmatic approach to capturing real-world practice across diverse NHS settings. Its design enables direct comparison with the first audit cycle, providing valuable longitudinal insight into progress over time. However, several limitations should be acknowledged. The retrospective nature of the study means it is dependent on the accuracy and completeness of electronic documentation, and some patient discussions may have occurred without being recorded. Only individuals who accessed secondary-care audiology or ENT services will be included, potentially underrepresenting those facing barriers in primary care or other community pathways. Certain regions or demographic groups may therefore be less represented. In addition, gender identity will be based on NHS-recorded sex data, and the absence of routinely performed speech audiometry in many centers limits evaluation of full NICE candidacy criteria. There may be local variations in practice or the structure of networks that affect referrals for a particular hospital or group of hospitals, which are not appreciated on a national scale, and therefore there may be further predictors of discussion or referral that this study fails to capture. Ethnicity data may also be impacted by the accuracy and quality of coding, which may not be evenly distributed across groups [20].

Despite these constraints, CIRCA-2 will provide the most comprehensive national overview to date of adult CI referral behavior, highlighting any persistent inequalities and modifiable service factors. The findings will directly inform national strategies to optimize referral pathways, strengthen awareness initiatives, and promote equitable access to hearing-restoration interventions across the United Kingdom.

Results of the study will be disseminated by presentation at national/international meetings and publication in peer-reviewed journals. Publication and presentation will be on behalf of all collaborators following the same corporate authorship model as previous INTEGRATE studies (ie, all investigators, regional leads, site leads, and steering group members meeting the criteria for authorship above). A lay summary will be produced for patients and the public. Dissemination of findings will allow the opportunity to address inequalities and improve referral rates across the United Kingdom.

## Supplementary material

10.2196/94888 Multimedia Appendix 1 User guide for running British Cochlear Implant Group cochlear implant referral report on Auditbase for the CIRCA INTEGRATE audit.

10.2196/94888 Multimedia Appendix 2 Outcome of the HRA decision tool showing that this study would not qualify as research and therefore does not require formal ethical approval. CIRCA: Cochlear Implant Referral Criteria Audit; HRA: Health Research Authority; NHS: National Health Service.

## Abbreviations

AB: Arthur Boothroyd Word Test
BAA: British Association of Audiology
BCIG: British Cochlear Implant Group
BSO: British Society of Otology
CI: cochlear implantation
CIRCA: Cochlear Implant Referral Criteria Audit
COACH: Comparing Cochlear Implants With Hearing Aids in Adults
ENT: ear, nose, and throat
INDICIA: Improving Navigation and Decreasing Inequality in Cochlear Implant Access
INTEGRATE: The UK ENT Trainee Research Network
NHS: National Health Service
NICE: National Institute for Health and Care Excellence
PMT: Project Management Team
STROBE: Strengthening the Reporting of Observational Studies in Epidemiology
