# Supplementary material for: Cochlear Implant Referrals in Adults With Severe-to-Profound Hearing Loss in the United Kingdom (CIRCA-2): Protocol for a Repeat National Multicenter Retrospective Observational Study
Source: JMIR Res Protoc. 2026 Oct 13;15:e94888. doi: 10.2196/94888 (PMC13660863; doi:10.2196/94888)
Supplement: Supplementary file 1 [file resprot-v15-e94888-s001.pdf]

User guide for running BCIG CI referral report on Auditbase for the CIRCA INTEGRATE audit

This guide provides information on how to set up and run the BCIG CI Referral Report. It includes minimum system requirements, details on how to set up the report on your local clinical database, step-by-step instructions on how to run the report, and how to export the data. The BCIG CI Referral Report was developed in collaboration with key NHS Audiology clinics, Auditdata and Cochlear; it is now supported by all four cochlear implant manufacturers (Advanced Bionics, Cochlear, MED-EL and Oticon Medical). The purpose of the report is to identify potential cochlear implant (CI) candidates based on their latest audiometric thresholds, in accordance with the NICE TA566 (2019) guidance on cochlear implants for children and adults with severe to profound deafness

This guide has been prepared by INTEGRATE, based on the BCIG CI referral report guidance with some amendments to make it relevant to this audit.

Top tips before you get started

1. Identify the audiologist in your department with admin rights on Auditbase. This person will be needed to set up the parameters that the Report scrapes from Auditbase, as only someone with admin rights can make edits like this in Auditbase.
2. Identify the Cochlear Implant Champion in your audiology department. This person can be helpful in data entry and may know the cochlear implant referral pathway in more detail.
3. A common setback is delays with IT. Be aware that you may need to involve your IT team in two of the key early steps of setting up the Report. The earlier you can involve them the better. Specifically, they may need to assist you with:
    a. Creating the “CI Referral Report” folder in your PC’s C Drive
    b. Permission to save files in this CI Referral Report folder (the two .rpt files required to run the report - “Cochlear Patient Decode Report.rpt” and “CI referral report.rpt”
4. When setting up the parameters in Auditbase, you will need to find certain parts of Auditbase, such as “List for report parameters in global definitions” (see page 9 of the instructions, where the search function is used in Auditbase). Depending on your version of Auditbase, these may be in slightly different places, and you may not have a search function. Rest assured they are there; it just may need a minute of clicking through the dropdown options to find them.

System requirements
Auditbase (provided by Auditdata) clinic management software version **5.2 or higher** is required to run the BCIG CI Referral Report. The BCIG CI Referral Report is available in Auditbase version **6.0.2 or higher as a standard report**. For **earlier versions** of Auditbase, the BCIG CI Referral Report needs to be **uploaded** by the clinic to the 'User Reports' section of their Auditbase database. Note Steps in this guide may differ depending on which version of Auditbase is currently used by the clinic.

System administration
The user may require computer system administration rights to upload the BCIG CI Referral Report to Auditbase. Alternatively, this can be supported by the clinic's IT department. The user will require Auditbase system **administration rights** for the initial set-up of the report (for versions below 6.0.2).

The report is **only available on the computer it was uploaded** on in some Trusts.

Report search criteria
The BCIG CI Referral Report identifies adult patients (≥ 18 years old) within a clinic's Auditbase database that meet the audiometric criteria for CI, as per NICE TA566 (2019) guidance on cochlear implants for children and adults with severe to profound deafness:

- Pure-tone audiometric thresholds equal to or greater than 80 dBHL at 2 or more frequencies (500 Hz, 1000Hz, 2000Hz, 3000Hz and 4000Hz)

The report uses the latest audiogram saved for a patient in Auditbase. Full details on the report search criteria and data fields can be found in Auditdata's BCIG CI Referral Report technical guide. Note: If the audiometric dataset for a patient is incomplete, the report may not identify this patient. The BCIG CI Referral Report identifies patients based on their audiometric thresholds. Not all patients identified by the report will be eligible for cochlear implantation. Suitability for cochlear implantation can only be determined once a patient has been assessed by a CI clinic

Report output

Results from the BCIG CI Referral Report are exported in an Excel spreadsheet and can be exported in three formats. This audit uses the internal format. All data must be completely anonymised prior to sending to the Project Management Team.

All formats of the exported data include the following information:

- Patient code –an automatically generated ID number allowing the clinic to cross reference data across Internal and External formats of the report. This ID code will remain assigned to a specific patient. However, if the patient is deleted from the clinic's Auditbase database, the code may be reassigned.
- Date of audiogram
- Parameter outcome – see section on Parameter set up for more details
- Air conduction thresholds – left and right ears
- Bone conduction thresholds – left and right ears
- Average air-bone gap – left and right ears
- Arthur Boothroyd (AB) word score – see section on AB word testing set-up and input
- Date of last fit - The latest hearing aid settings stored on Auditbase
- Hearing device – latest device listed in the stock module

Report set-up

For versions of Auditbase inclusive of 5.2 to 6.0.1, the BCIG CI Referral Report needs to be set up in the clinic's Auditbase system by **a system administrator**. For clinics using version 6.0.2 or higher, please *skip to 'ODBC set up' (page 9)*. This section will take you through how to upload the BCIG CI Referral Report in Auditbase and set up functions that allow for data to be shown on the exported report.

Click on 'System settings'

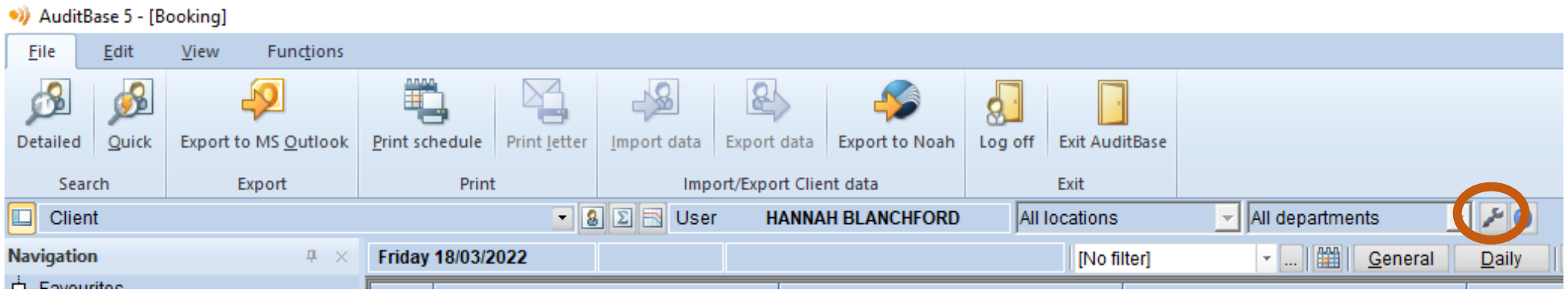

Click **Open system administration**

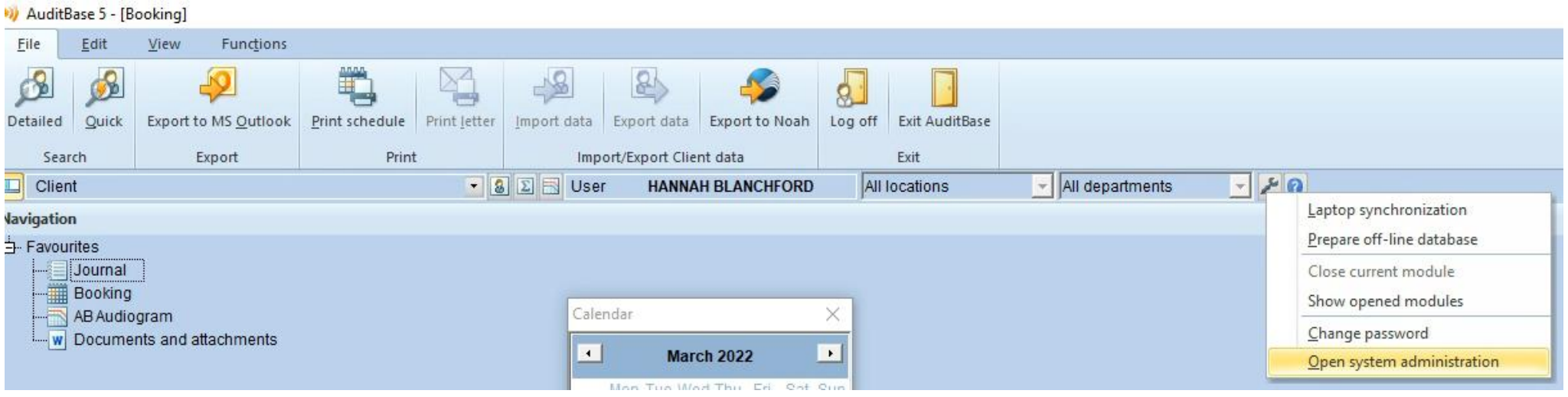

You will be asked to log in as a system administrator. **Use your local system administrator login**

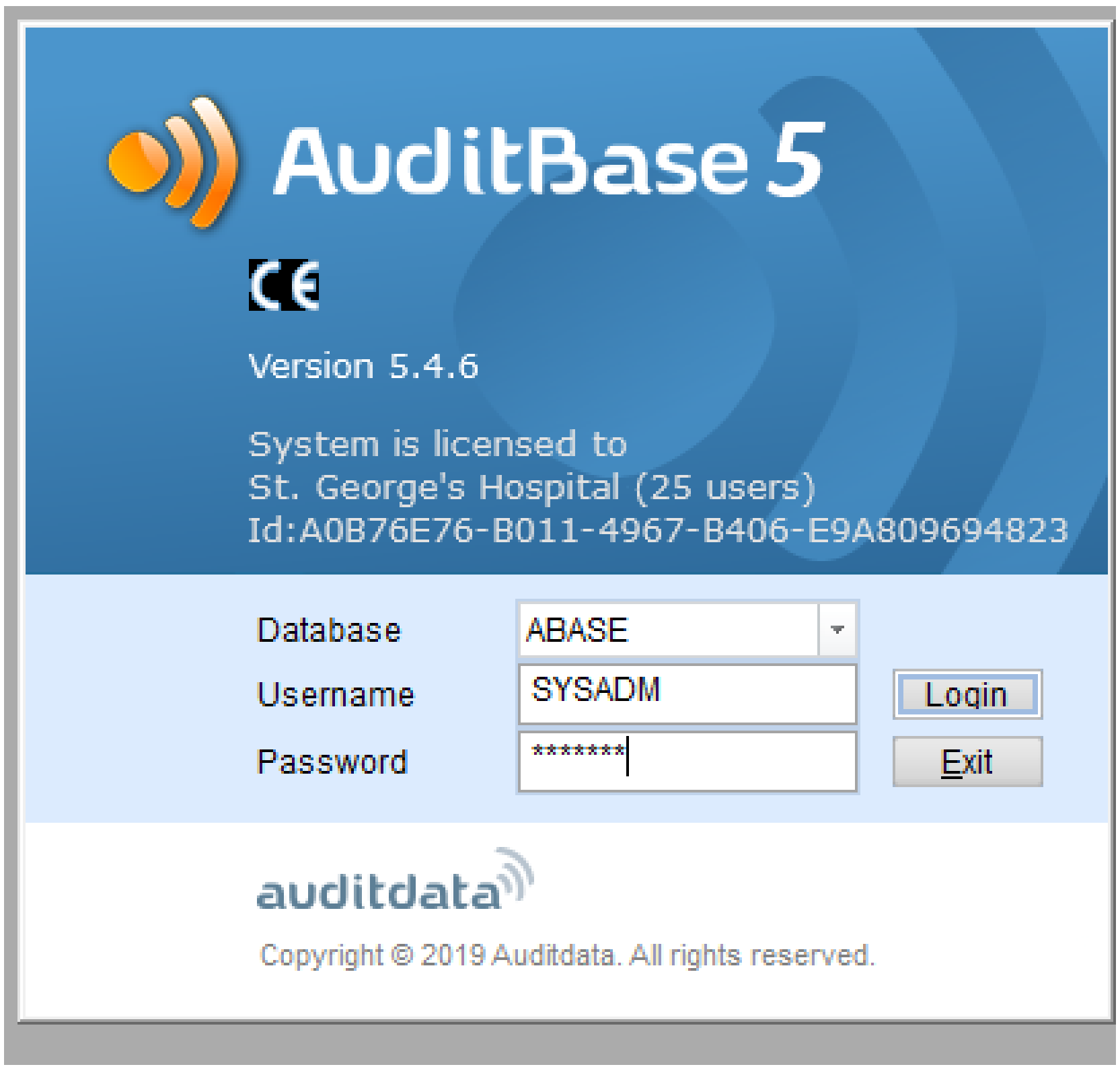

Click on **User and workstation settings**, then click **Other**

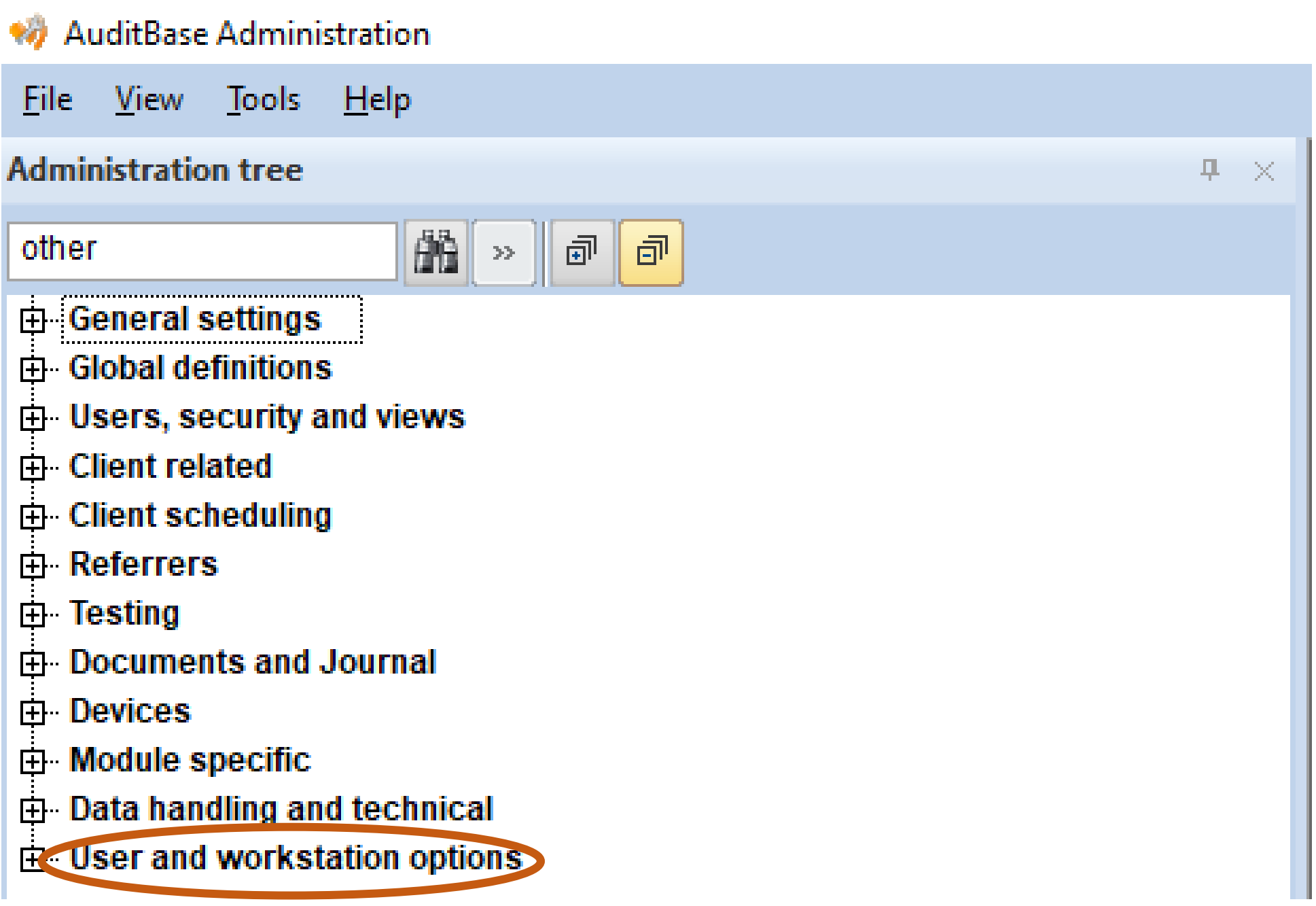

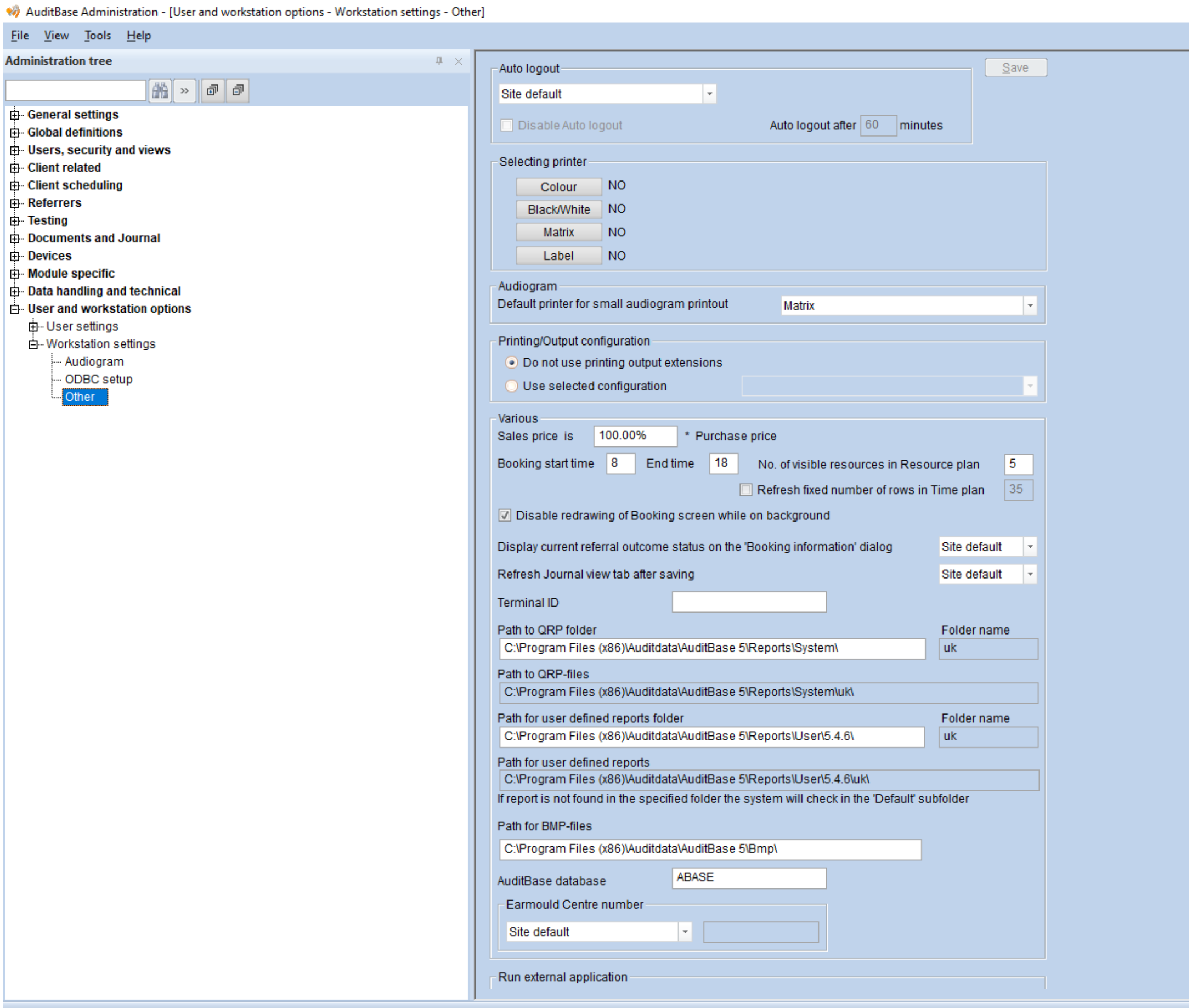

Copy the location text under **Path for user defined reports. Ensure you select the correct version of your local Auditbase**

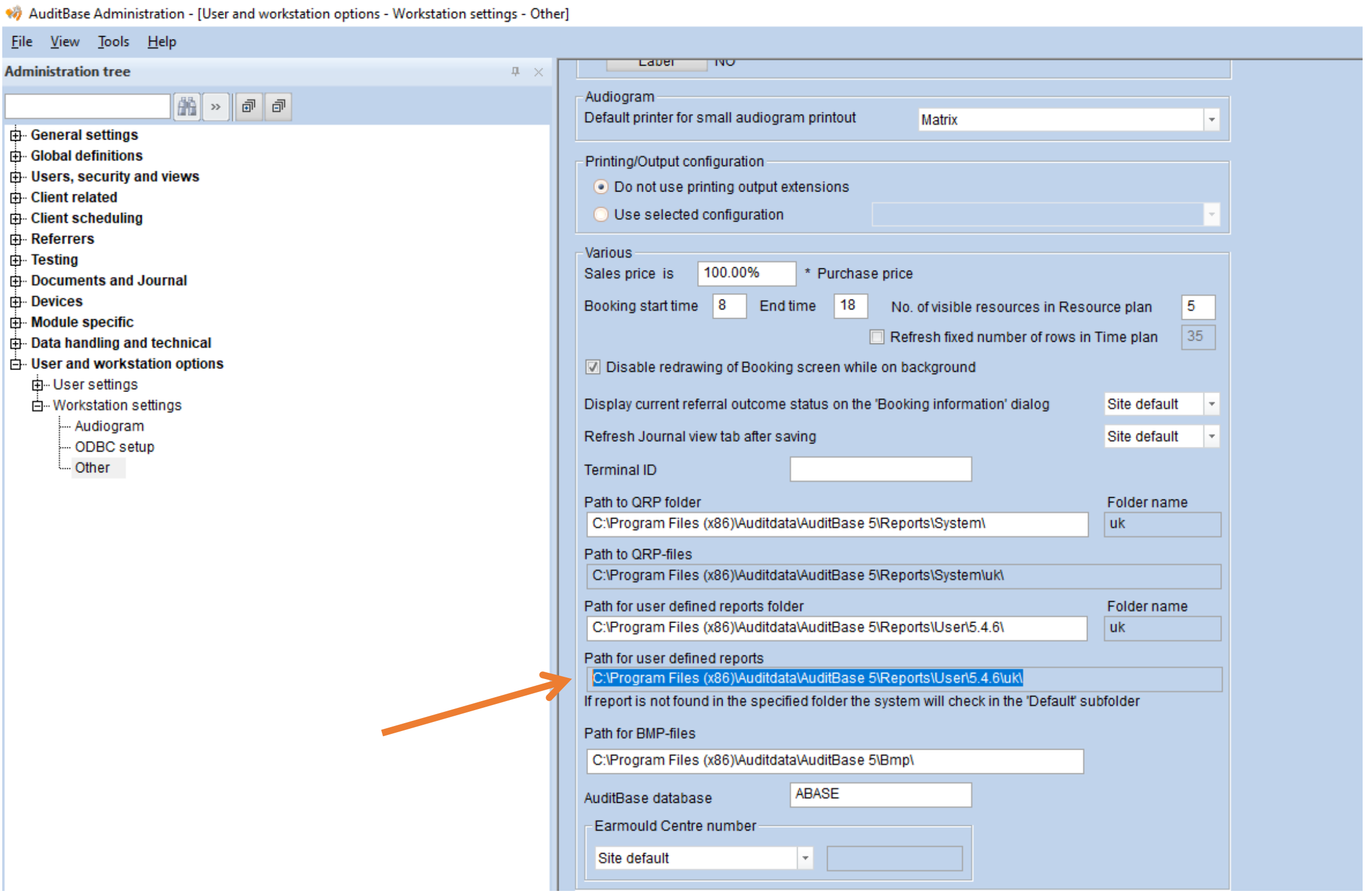

You may require special administrator rights to create a new folder in the C: drive, **or may need to involve your IT department in doing so. If involving IT in the creation of the folder, it is important to flag that you will need permission to save files into this folder for the next step of the process**. The following instructions work if your crystal reports are located in the C: drive. File paths may differ slightly across centres depending on how your database is set up. Be sure to create your 'CI referral report' folder in the same location that is listed under **Path for user defined reports**

Using file explorer, go to the location listed under **Path for user defined reports**:

C drive
>Program Files (x86)
>Auditdata
>Auditbase 5
>Reports
>User
>5.4.6
>Uk

Create a new folder labelled **CI Referral Report**

The path will look like this:
C:\Program Files (x86)\Auditdata\AuditBase 5\Reports\User\5.4.6\uk\CI Referral Report

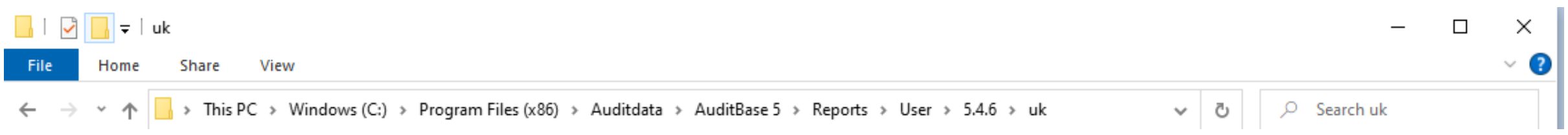

Locate **the CI Referral Report** and **Patient decode report** files in your emails from INTEGRATE.

Click **Save as on the attachments**

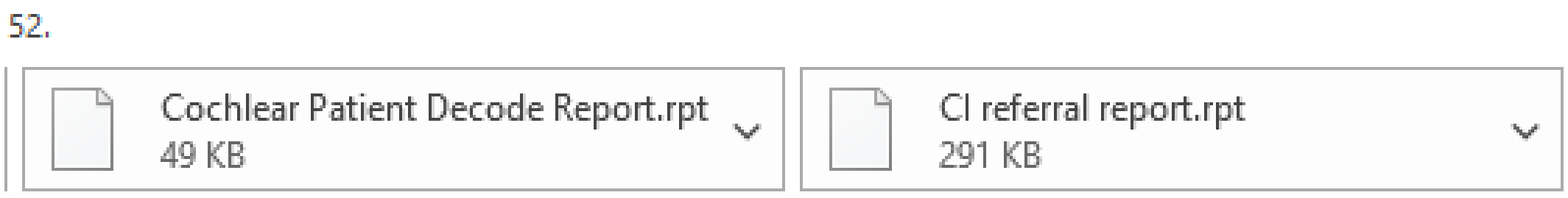

Save the **CI Referral Report.rpt** and **Patient Decode report.rpt** files into the newly created folder (C:\Program Files (x86)\Auditdata\AuditBase 5\Reports\User\5.4.6\uk\CI Referral Report). You may require special administrator rights to save files into the new folder, **or may need to involve your IT department in doing so (as highlighted above).**

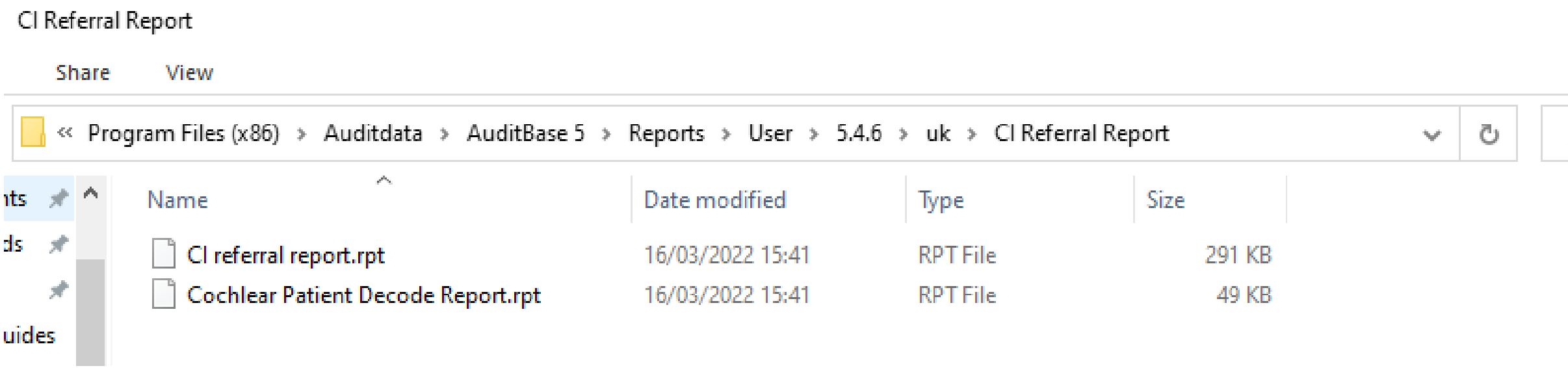
CI Referral Report
Share
View
Program Files (x86) › Auditdata › AuditBase 5 › Reports › User › 5.4.6 › uk › CI Referral Report
Name
Date modified
Type
Size
CI referral report.rpt
16/03/2022 15:41
RPT File
291 KB
Cochlear Patient Decode Report.rpt
16/03/2022 15:41
RPT File
49 KB

ODBC set-up

In Auditbase version 5.2, click **ODBC setup** under the Workstation settings

Click **enable driver** and **Save**

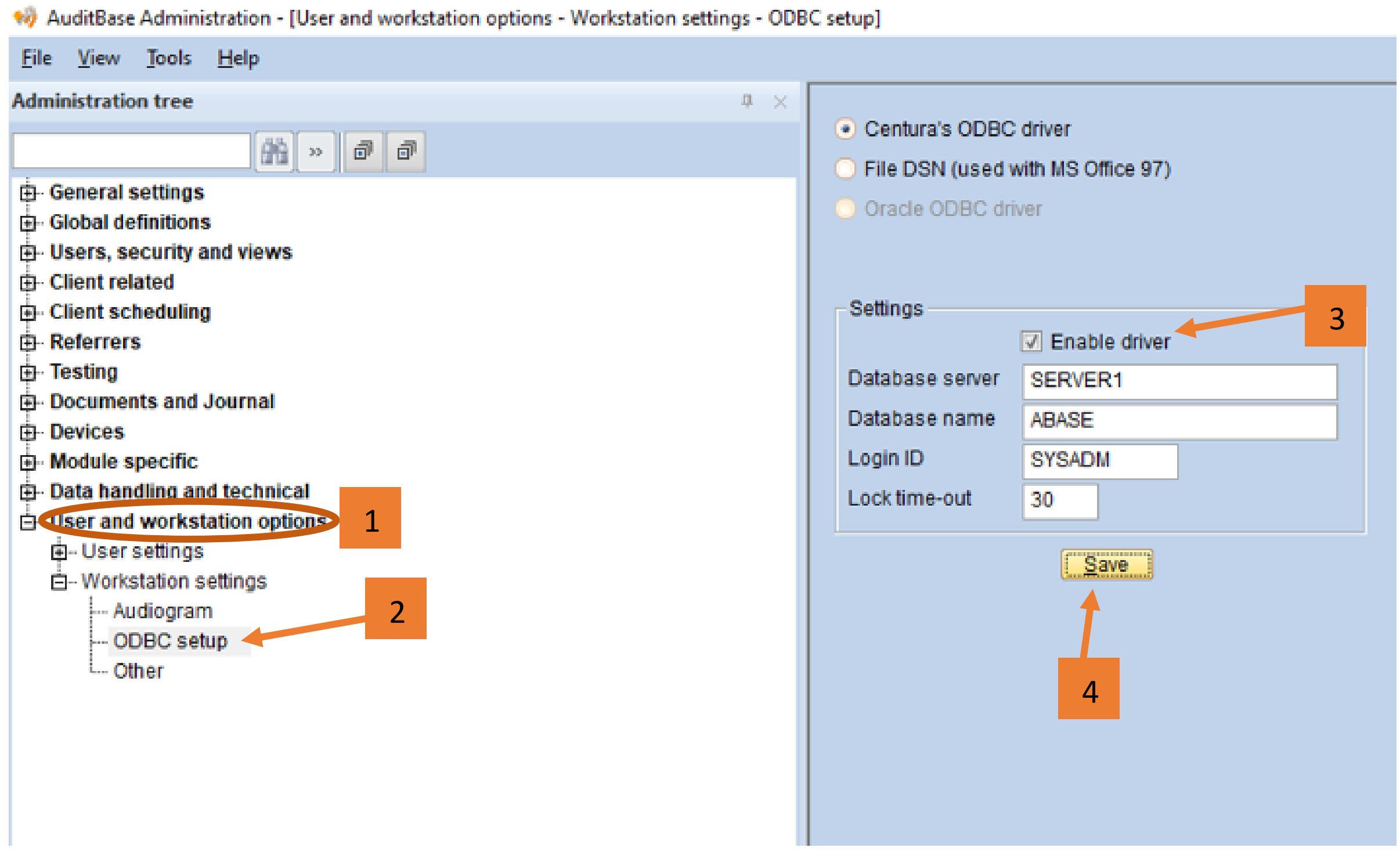

In Auditbase version 6.0.2 (and higher), click **workstation settings**, then click **Printing and reports**

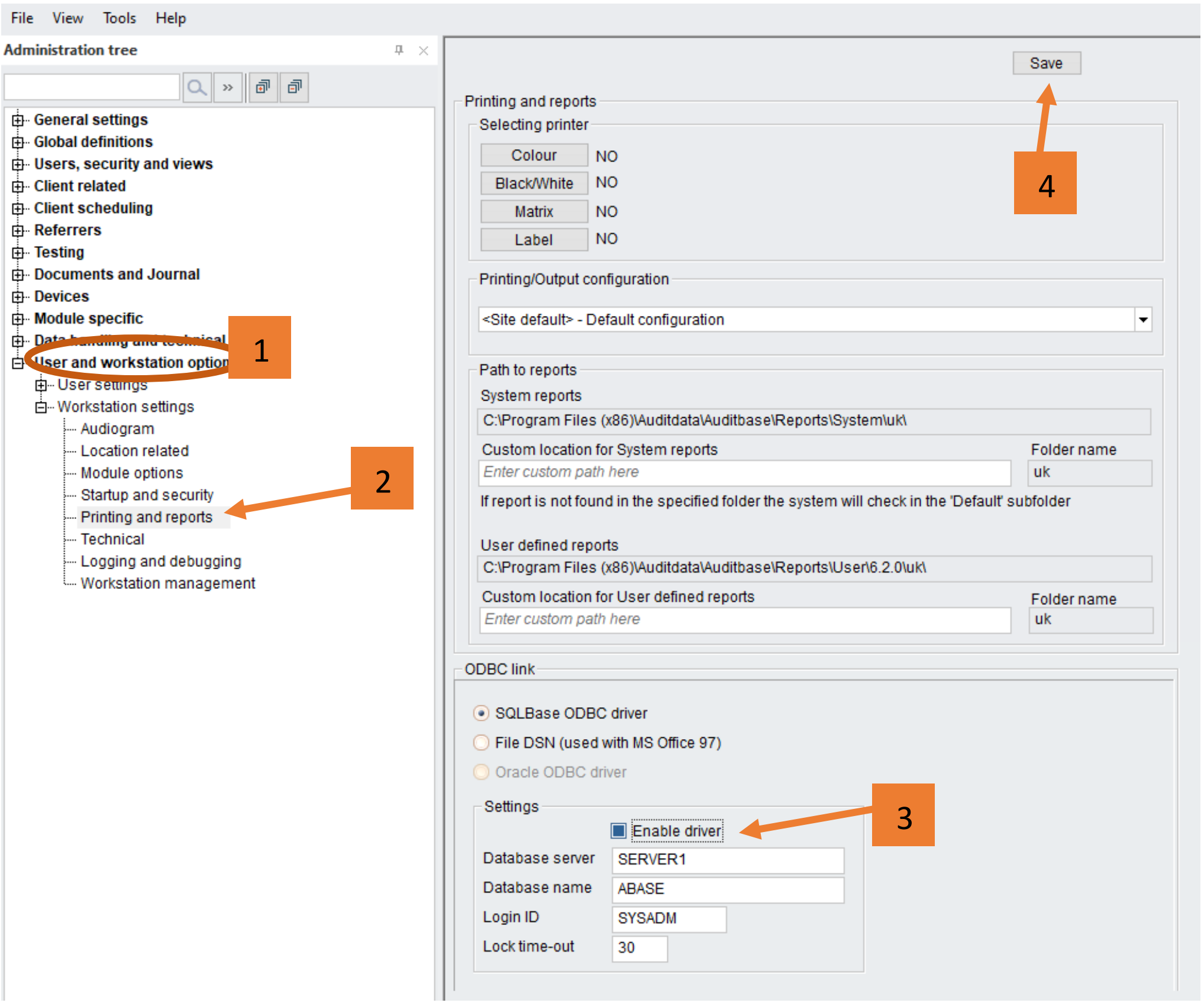

Ensure you click **enable driver** and then click **Save** (at the top of screen). **Log off auditbase, then on again.**

Parameter set-up

As a system administrator, search for **definitions** in the administration tree

Click on **Lists for report parameters** (or alternative, such as client alert and parameters)

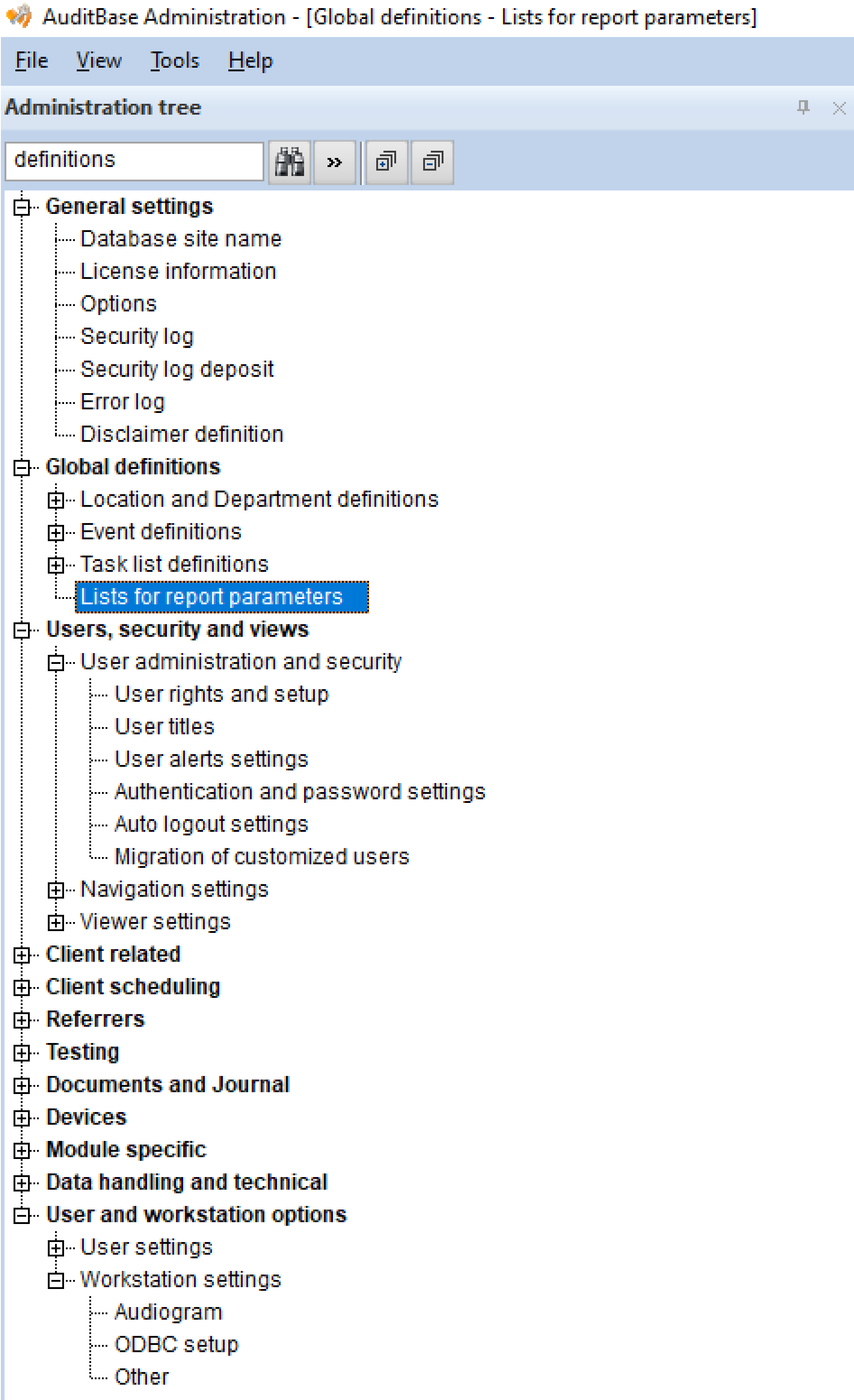

Click **New**

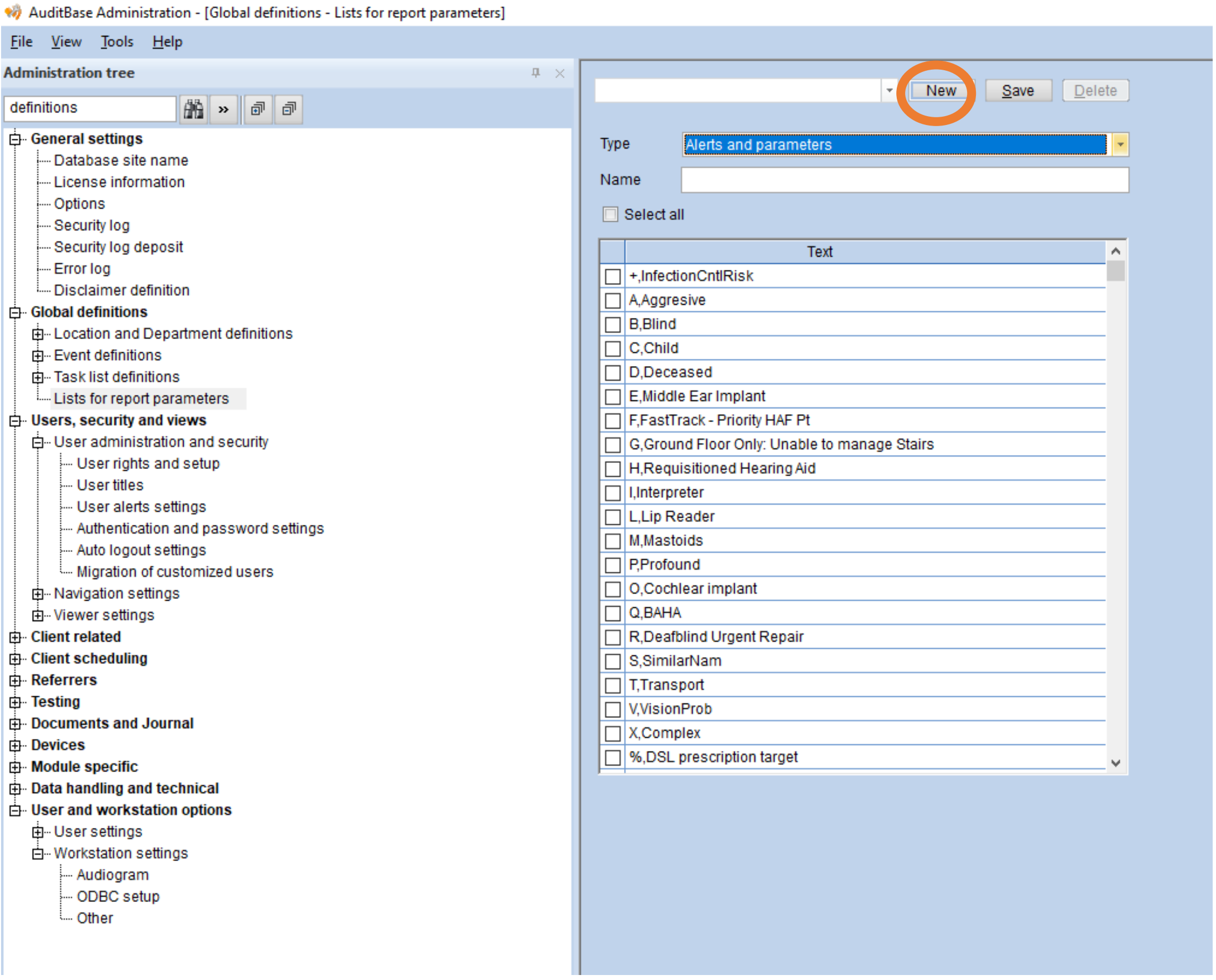

Click Alerts and parameters in the dropdown menu

Add the following 7 parameters and click Save after each parameter is added

- CI1 – referred for CI
- CI2 – not referred – unsuitable/ outside of criteria
- CI3 – not referred – discussed and further assessment needed
- CI4 – not referred – patient declined
- CI5 – CI to be discussed
- CI6 – under assessment
- CI patient

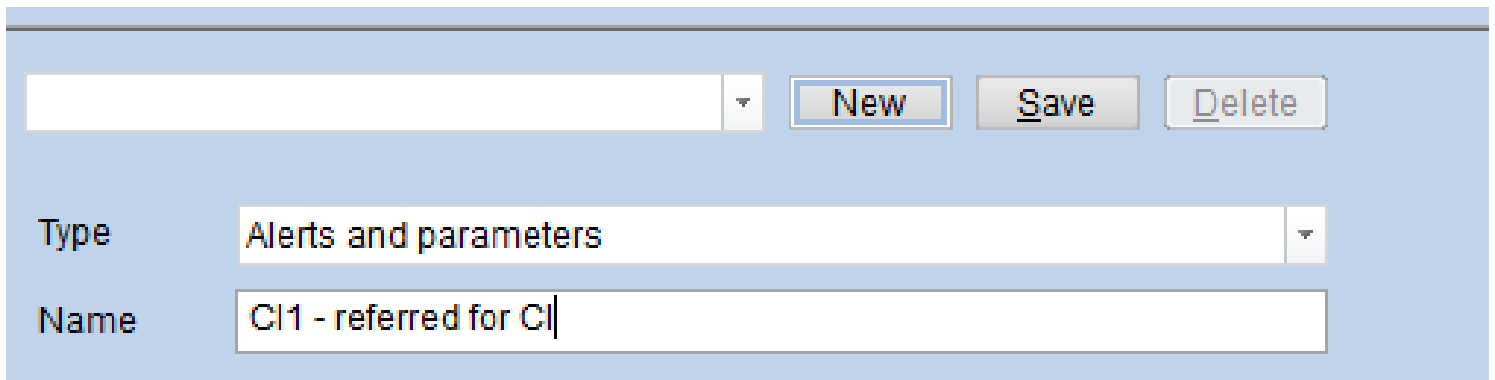

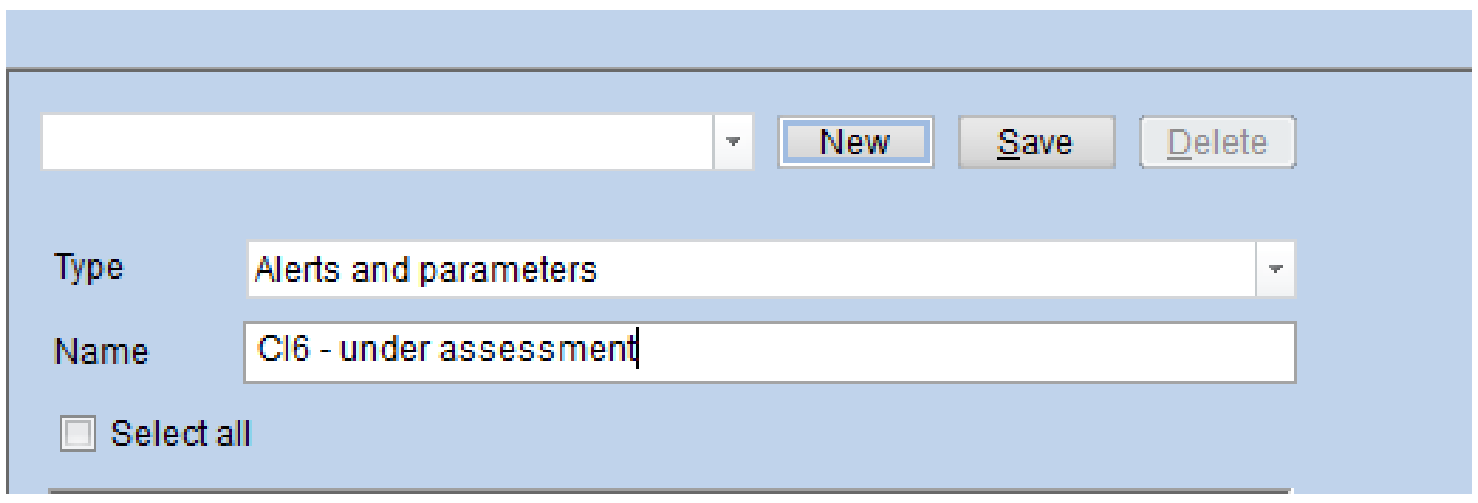

AB word testing set-up

Click the administration tree, search **'other speech'**

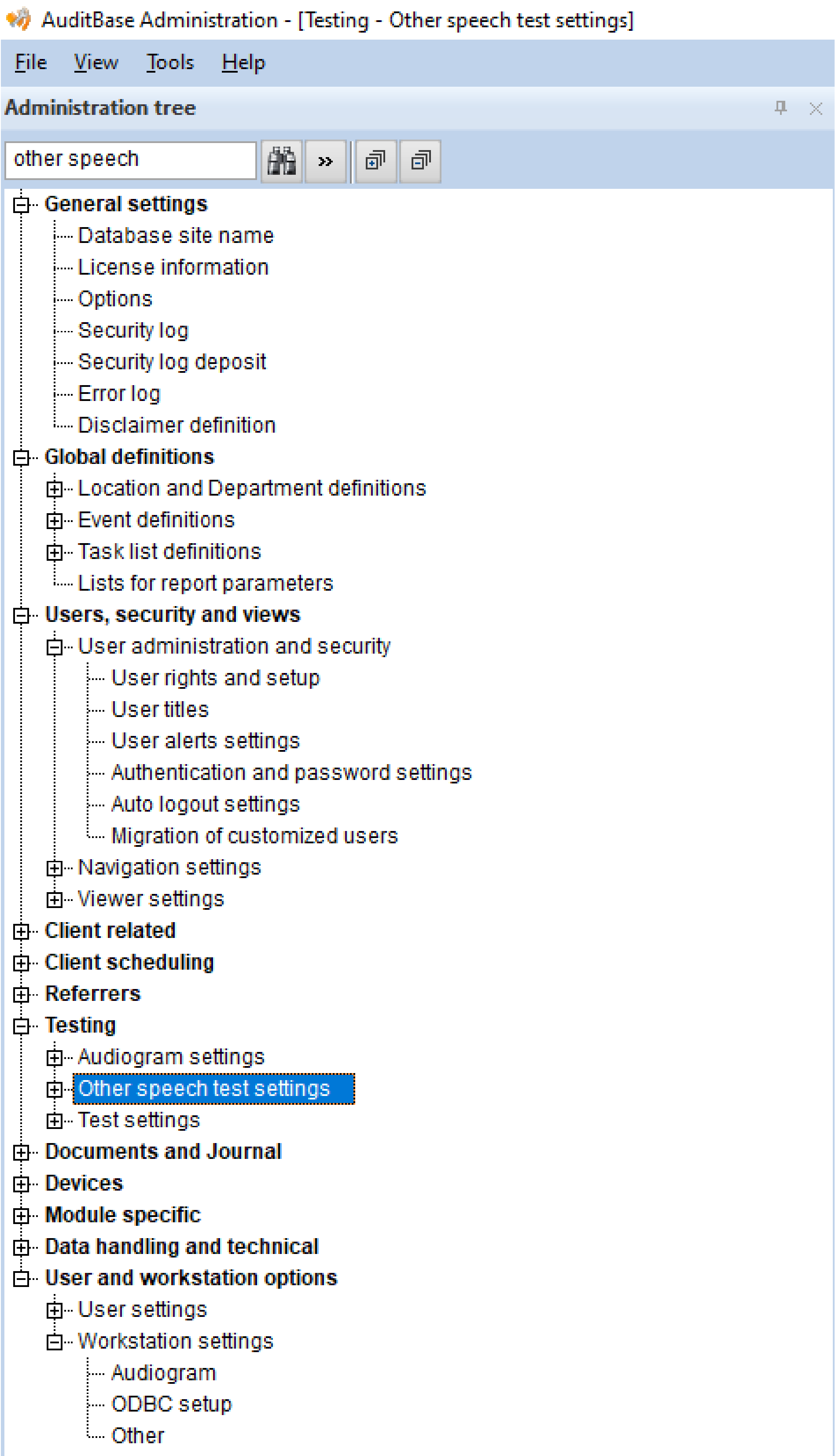

Ensure AB word list is in the list of speech tests

If not, **click New** and add it

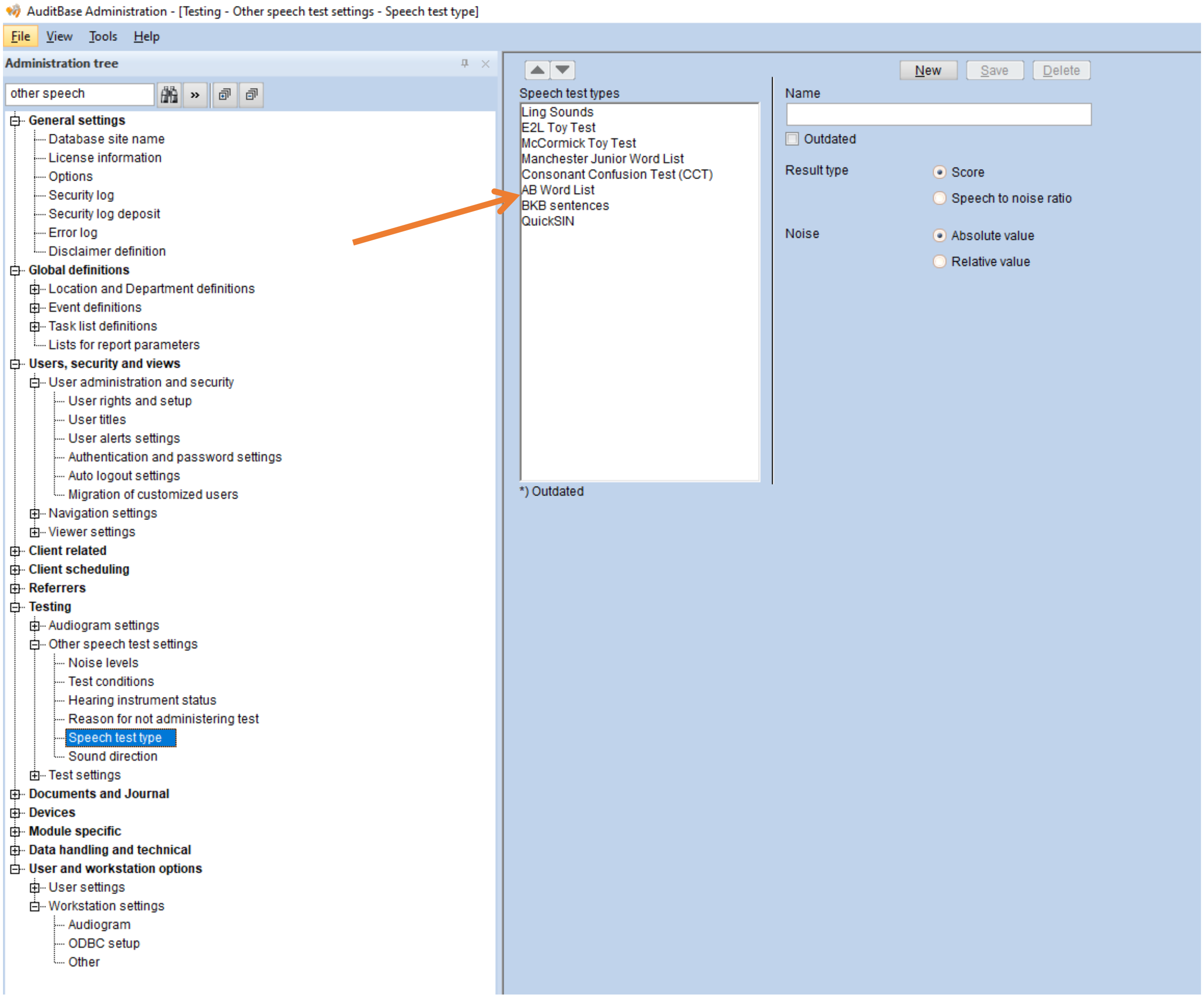

Running the report

Click on **clinic management**

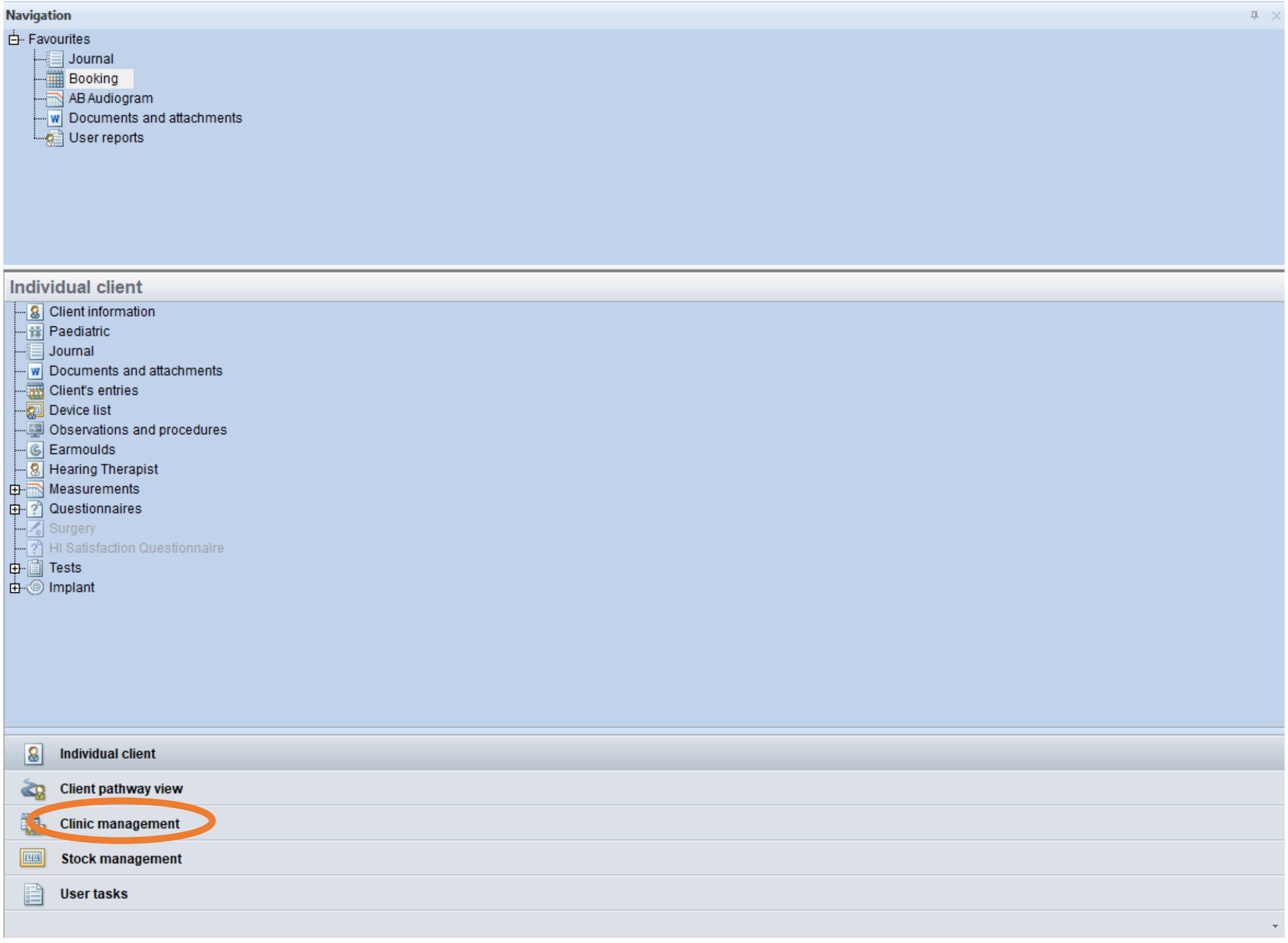

Select **User reports**

In Auditbase 5.2, select **CI referral report**

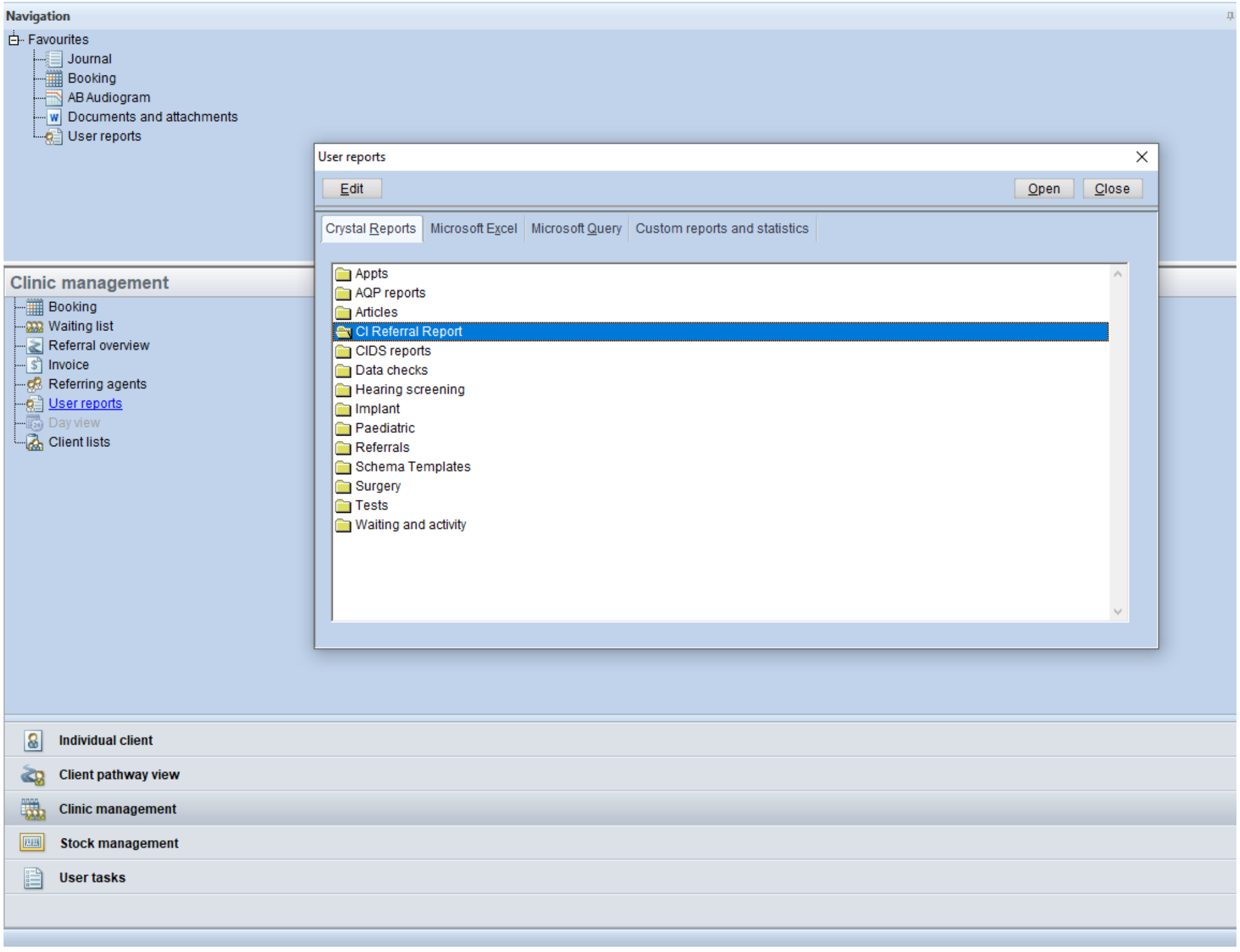

In Auditbase 6.0.2 (and higher), click **Implant**, then **Patient overviews**, then **CI referral report.rpt**

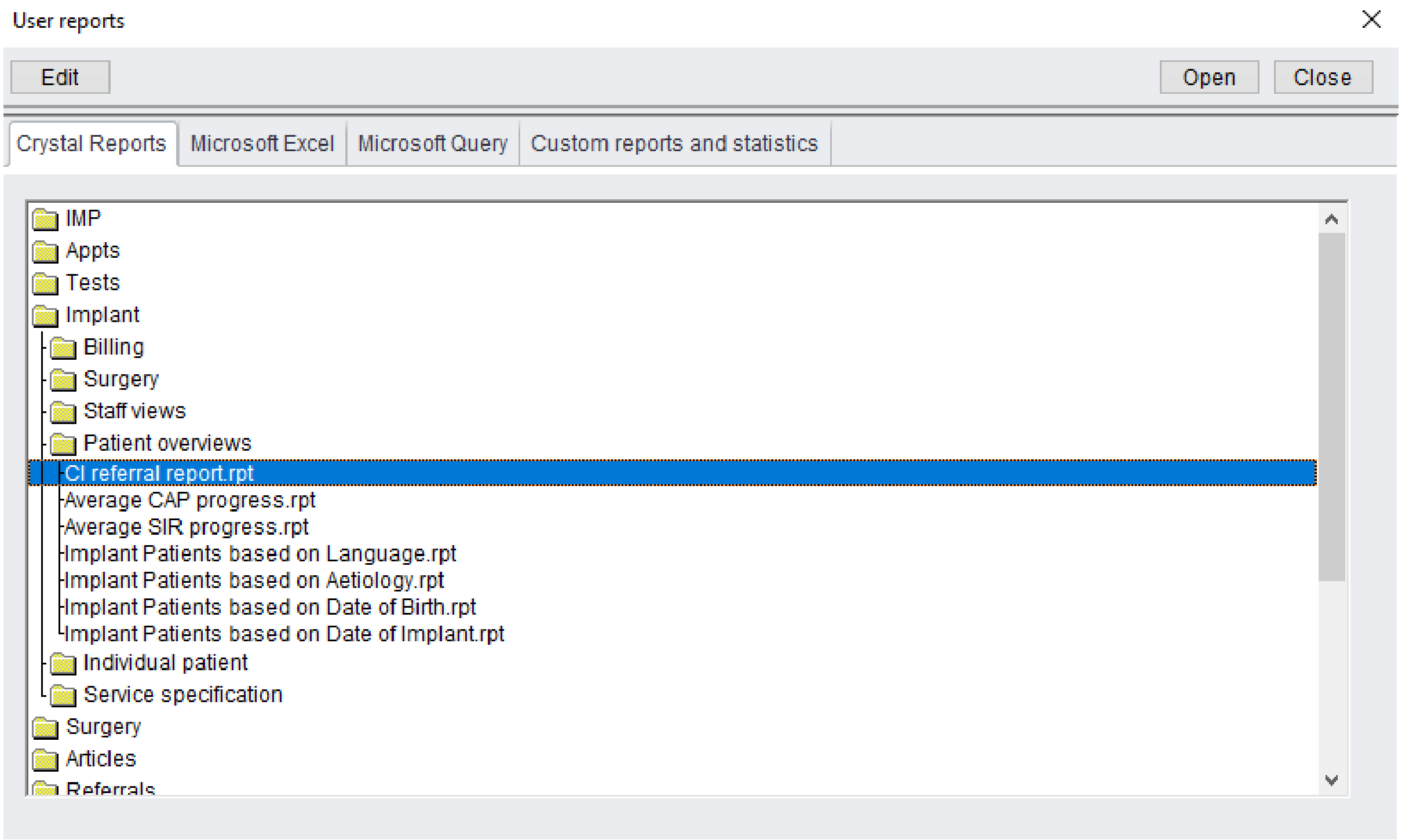

Both versions

Select the date range 2021-7-1 to 2021-12-31

Select include reports

Select internal report

Let the report run. This could take up to 8 minutes

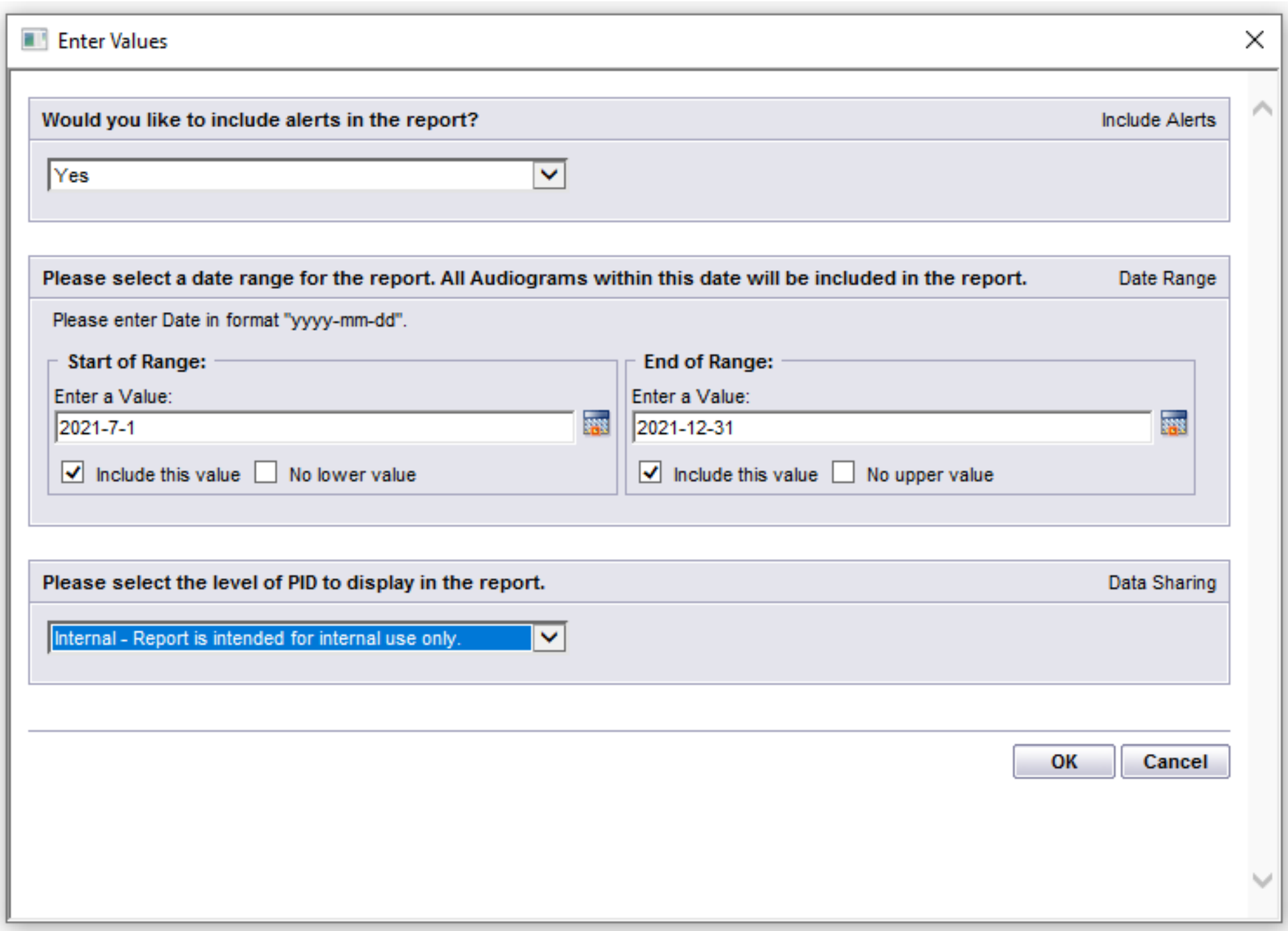

Select Export result from the top left hand corner

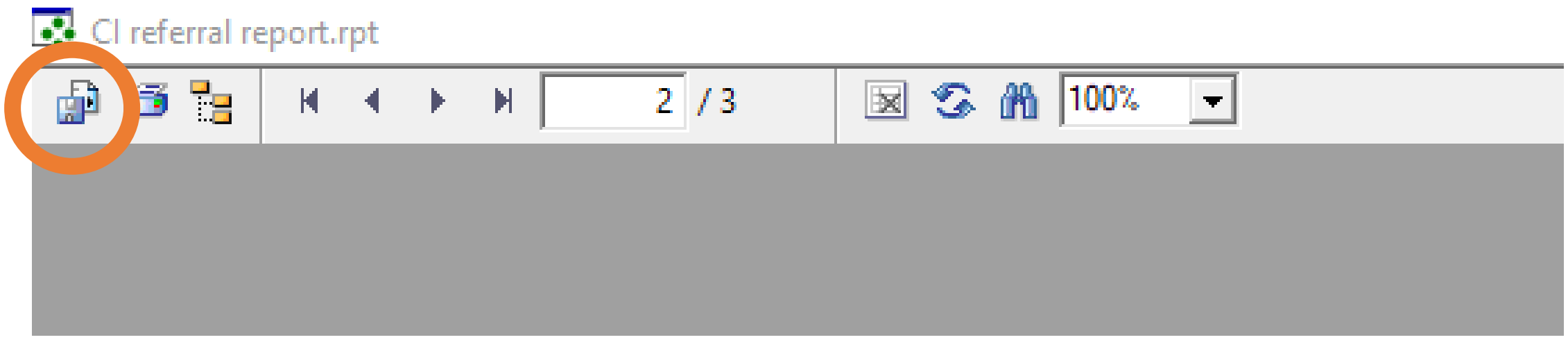

Select Microsoft Excel 97-2000 (XLS)

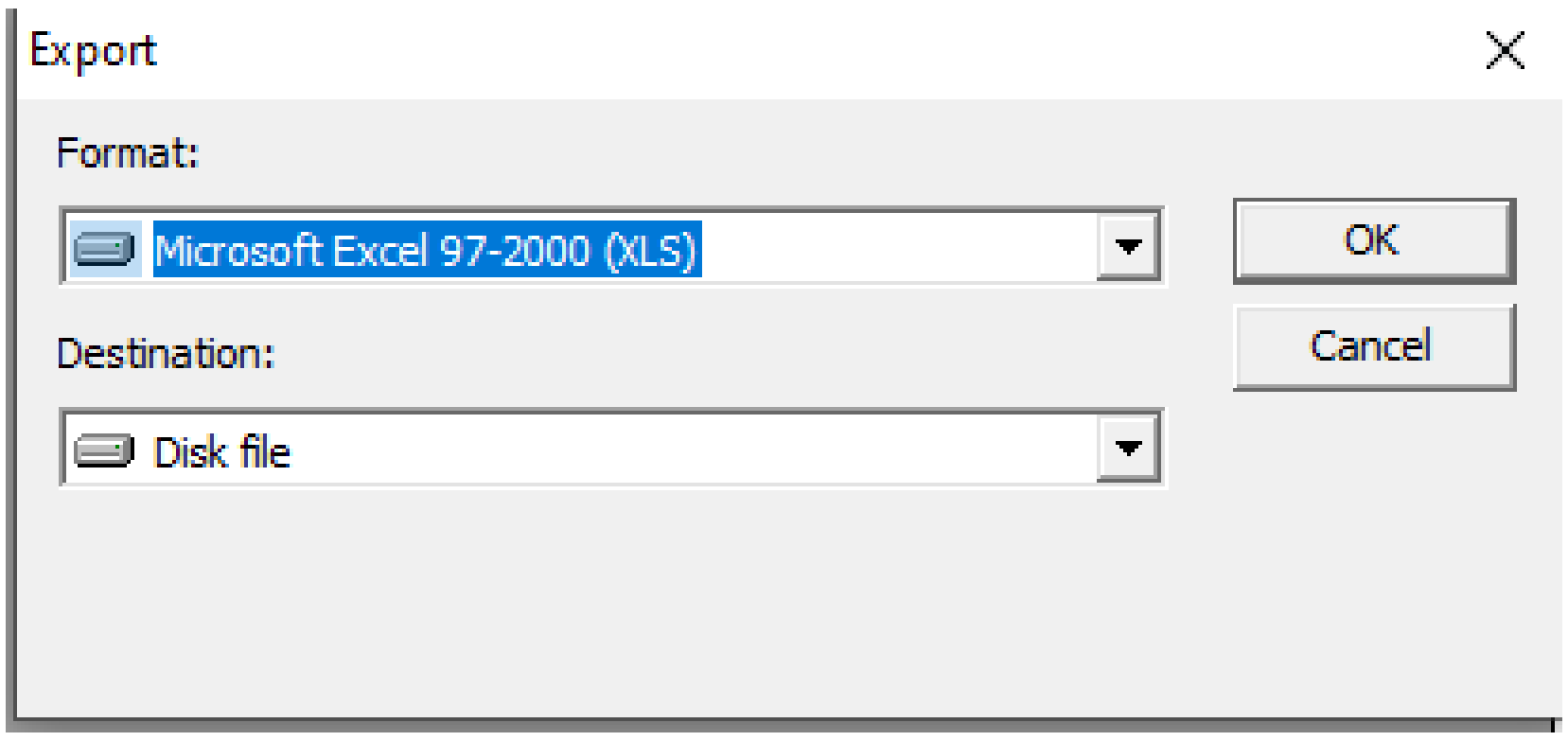

Click OK

Select the following options

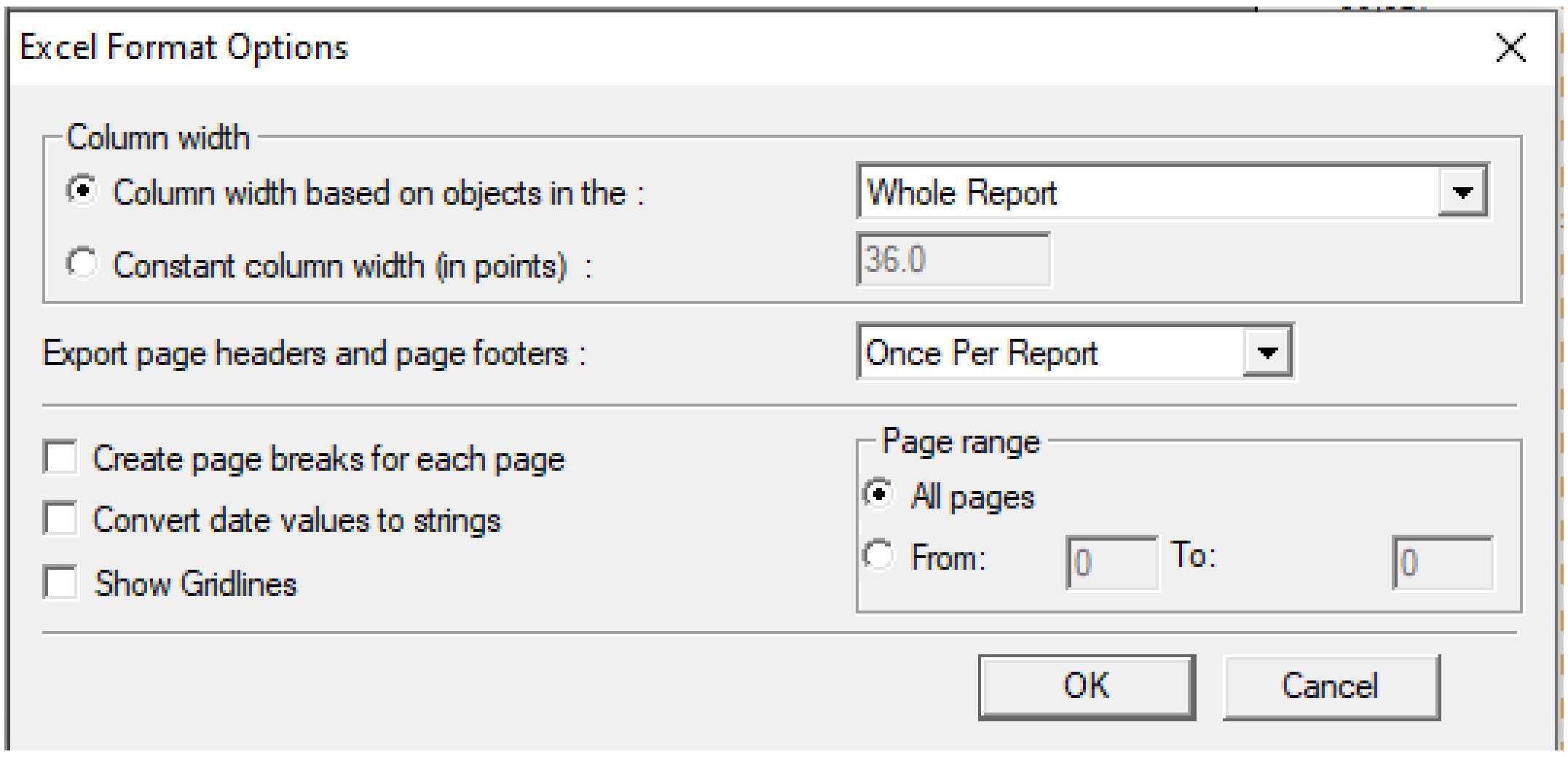

Save to your preferred location
Open the file to access your datasheet

Follow instructions provided by INTEGRATE for the next steps

References

British Cochlear Implant Group. BCIG CI Referral Report user guide (2020)
